# Obesity reprograms the pulmonary polyunsaturated fatty acid-derived lipidome, transcriptome, and gene-oxylipin networks

**DOI:** 10.1016/j.jlr.2022.100267

**Published:** 2022-08-24

**Authors:** Rafia Virk, Nicole Buddenbaum, Abrar Al-Shaer, Michael Armstrong, Jonathan Manke, Nichole Reisdorph, Selin Sergin, Jenifer I. Fenton, E. Diane Wallace, Brandie M. Ehrmann, Hannah B. Lovins, Kymberly M. Gowdy, M Ryan Smith, Gregory J. Smith, Samir N.P. Kelada, Saame Raza Shaikh

**Affiliations:** 1 Department of Nutrition, Gillings School of Global Public Health and School of Medicine, The University of North Carolina at Chapel Hill, Chapel Hill, NC, USA; 2 Department of Pharmaceutical Sciences, University of Colorado Denver Anschutz Medical Campus, Aurora, CO, USA; 3 Department of Food Science and Human Nutrition, College of Agriculture and Natural Resources and College of Osteopathic Medicine, Michigan State University, East Lansing, Michigan, USA; 4 Department of Chemistry, University of North Carolina at Chapel Hill, Chapel Hill, NC, USA; 5 Division of Pulmonary, Critical Care and Sleep Medicine, The Ohio State University, Columbus, OH, USA; 6 Division of Pulmonary, Allergy, Critical Care, and Sleep Medicine, Department of Medicine, Emory University, Atlanta, GA, USA; 7 Atlanta Department of Veterans Affairs Medical Center, Decatur, GA, USA; 8 Marsico Lung Institute, University of North Carolina at Chapel Hill, Chapel Hill, NC, USA; 9 Department of Genetics, University of North Carolina at Chapel Hill, Chapel Hill, NC, USA

**Keywords:** arachidonic acid, high-fat diet, oxylipins, inflammation, nutrition, omega-3 fatty acids, prostaglandins, transcriptional regulation, triglycerides, glycerophospholipid metabolism, AA, arachidonic acid, ALI, acute lung injury, BALF, bronchoalveolar lavage fluid, BH, Benjamini-Hochberg, DHA, docosahexaenoic acid, EPA, eicosapentaenoic acid, FAME, fatty acid methyl ester, HFD, high-fat diet, LC-PUFA, long-chain polyunsaturated fatty acid, PS, phosphatidylserine, SPMs, specialized pro-resolving mediators

## Abstract

Obesity exacerbates inflammation upon lung injury; however, the mechanisms by which obesity primes pulmonary dysregulation prior to external injury are not well studied. Herein, we tested the hypothesis that obesity dysregulates pulmonary PUFA metabolism that is central to inflammation initiation and resolution. We first show that a high-fat diet (HFD) administered to C57BL/6J mice increased the relative abundance of pulmonary PUFA-containing triglycerides and the concentration of PUFA-derived oxylipins (particularly prostaglandins and hydroxyeicosatetraenoic acids), independent of an increase in total pulmonary PUFAs, prior to onset of pulmonary inflammation. Experiments with a genetic model of obesity (*ob/ob*) generally recapitulated the effects of the HFD on the pulmonary oxylipin signature. Subsequent pulmonary next-generation RNA sequencing identified complex and unique transcriptional regulation with the HFD. We found the HFD increased pathways related to glycerophospholipid metabolism and immunity, including a unique elevation in B cell differentiation and signaling. Furthermore, we conducted computational integration of lipidomic with transcriptomic data. These analyses identified novel HFD-driven networks between glycerophospholipid metabolism and B cell receptor signaling with specific PUFA-derived pulmonary oxylipins. Finally, we confirmed the hypothesis by demonstrating that the concentration of pulmonary oxylipins, in addition to inflammatory markers, were generally increased in mice consuming a HFD upon ozone-induced acute lung injury. Collectively, these data show that a HFD dysregulates pulmonary PUFA metabolism prior to external lung injury, which may be a mechanism by which obesity primes the lungs to respond poorly to infectious and/or inflammatory challenges.

The worldwide prevalence of obesity has significantly increased over the last three decades (1, 2). Obesity, characterized by increased adiposity and chronic uncontrolled inflammation, increases the risk for a variety of complications such as cardiovascular diseases, nonalcoholic fatty liver disease, type 2 diabetes, hypertension, and cancers. Notably, there is considerable interest in understanding how obesity contributes to respiratory inflammation. Studies across model systems suggest that obesity contributes toward poor pulmonary outcomes (3, 4, 5, 6, 7, 8). This is particularly relevant with the current COVID-19 pandemic in which individuals with obesity present with more severe pulmonary complications in response to SARS-CoV-2 infection (9). However, a major limitation is that our understanding of the underlying mechanisms by which diet-induced obesity contributes toward dysregulated pulmonary inflammation remains unclear.

One set of factors that control susceptibility to pulmonary injury and inflammation in obesity are long-chain polyunsaturated fatty acids (LC-PUFAs) and their downstream oxylipins. These oxylipins include several families of molecules such as prostaglandins and leukotrienes, which are synthesized from the n-6 LC-PUFA arachidonic acid (AA) and have a critical role in the initiation of inflammation (10). Oxylipins also include specialized pro-resolving mediators (SPMs) and their metabolic intermediates that drive the resolution of inflammation and return damaged tissue to homeostasis. SPMs can be enzymatically synthesized from the n-3 LC-PUFAs, EPA, and DHA, in addition to synthesis from AA (10). Dysregulation of SPM metabolism is linked with the progression of multiple pulmonary disease contexts including asthma, COPD, and cystic fibrosis (11, 12, 13, 14).

There is compelling evidence from studies with mice and humans that obesity drives an imbalanced signature of PUFA-derived oxylipins. For instance, the AA-derived leukotriene B4 is elevated in obese mice and has a central role as a chemoattractant in white adipose tissue to promote chronic inflammation (15, 16). Additionally, SPMs and their intermediates or precursors are significantly lowered in response to obesity across a range of mouse tissues including white adipose tissue, liver, bone marrow, and spleen (17, 18, 19, 20, 21). In humans with obesity, leukocytes have a 4-fold increase in leukotriene B4 and decreased levels of EPA- and DHA-derived oxylipins (22). These results suggest the hypothesis, which was tested in this study, that obesity may also dysregulate the balance of PUFA-derived oxylipins in lung tissue. This imbalance in oxylipins can ultimately contribute to exacerbated pulmonary inflammation.

The first objective of this study was to test the hypothesis that obesity controls the pulmonary PUFA-containing lipidome prior to external lung injury. We first established that a high-fat diet (HFD) does not promote overt lung inflammation. We then used untargeted LC-MS to investigate how a HFD dysregulates the relative abundance of pulmonary total and PUFA-containing triglycerides, diglycerides, and differing phospholipid classes. These studies led to targeted LC-MS/MS to determine how HFD-induced obesity controls the concentration of PUFA-derived oxylipins in the absence of external lung injury. Experiments were also conducted with a genetic model of obesity to determine if the effects were specific to a HFD-induced model of obesity on the pulmonary oxylipin profile. Subsequently, next generation RNA sequencing was employed to investigate how a HFD, in the absence of external lung injury, impairs the underlying transcriptome that is related to PUFA metabolism and immunity. We also used a computational tool (xMWAS) to integrate the lipidomic and transcriptomic analyses, which reveal novel networks between specific oxylipins and genes in response to a HFD (23). Finally, the basic hypothesis that a HFD dysregulates the pulmonary PUFA-derived oxylipin profile and accompanying inflammation was tested using an ozone-induced acute lung injury (ALI) model.

## Materials and methods

### Animal models and diets

All murine experiments followed the NIH Guide for the Care and Use of Laboratory Animals and were reviewed and approved by The University of North Carolina at Chapel Hill and The Ohio State University IACUC for euthanasia and humane treatment. Euthanasia relied on CO_2_ or isoflurane inhalation followed by cervical dislocation or exsanguination. Mice for the ALI model studies were euthanized by urethane followed by exsanguination. C57BL/6J male mice were purchased from Jackson Laboratories. At 6 weeks of age, mice were fed lean control (10% kcal from fat) or HFD (60% kcal from fat) for 15 weeks (Research Diets, New Brunswick, NJ). *Ob/ob* mice, a genetic model of obesity driven by leptin deficiency, were purchased from Jackson laboratories at age 7 weeks and were fed a normal chow diet for an additional 3 weeks.

### Metabolic profiling

Glucose tolerance tests and fasting glucose/insulin measurements were conducted after 14 weeks of feeding lean diets or HFDs, as previously described (20). *Ob/ob* mice were also subjected to glucose tolerance tests at 9–10 weeks of age. Mice were fasted for 5 h prior to the establishment of baseline glucose values with a glucometer. For the glucose tolerance test, 2.5 g of dextrose (Sigma-Aldrich, St. Louis, MO) per kg lean mass was administered intraperitoneally. Insulin values were measured after a 5 h fast and quantified via ELISA (Crystal Chem, Elk Grove Village, IL) (20).

### qRT-PCR

qRT-PCR of lung inflammatory cytokines and chemokines were measured as previously described (24). Primers for qRT-PCR are the same as previously shown (24).

### Isolation of bronchoalveolar lavage fluid and analysis

Bronchoalveolar lavage fluid (BALF) was collected immediately following sacrifice. All lung lobes were lavaged 3 times with 1 ml volumes of saline each. The resulting lavage was centrifuged (1800 RPM, 6 min, 4°C), and an aliquot of the supernatant was removed for protein analysis using a Pierce BCA Protein-Assay Kit (Thermo Scientific, Hercules, CA). The cell pellets were suspended in 1 ml of red blood cell ammonium chloride potassium lysis buffer, vortexed, and incubated for 1 min. To stop the reaction, 4 ml of 1×PBS was added. The cells were then centrifuged again at 1800 RPM for 6 min at 4°C, the supernatant was aspirated, and 1 ml of 10% fetal bovine serum was added to the pellet of cells. Total cell counts in the BALF were obtained by manually counting with a hemocytometer (Hausser Scientific, Horsham, PA). Each sample (120 μl) was centrifuged onto slides using a Cytospin 4 (ThermoFisher, Waltham, MA) and subsequently stained with Diff Quik solution (ThermoFisher) for differential cell counts, with at least 200 cells counted from each slide.

### Untargeted LC-MS lipidome analysis

Mouse lungs were weighed into Eppendorf tubes. The lung tissues were mashed using a clean metal spatula prior to extraction. The samples were extracted using a liquid-liquid partition with water (250 μl), methanol (300 μl), and methyl tert-butyl ether. Avanti’s deuterated lipid mix, Equisplash, was used as an internal standard. The deuterated mix was spiked into the methanol at 1.5 μg/ml and used for extraction. The extracts were centrifuged at 20,000 rcf for 10 min to facilitate phase separation. The top layer was removed, dried, and reconstituted in 150 μl of isopropanol for analysis.

Analysis was performed using a Thermo Q Exactive Plus coupled to a Waters Acquity H-Class LC. A 100 mm x 2.1 mm, 2.1 μm Waters BEH C18 column was used for separations. The following mobile phases were used: A- 60/40 ACN/H20, B- 90/10 IPA/ACN; both mobile phases had 10 mM ammonium formate and 0.1% formic acid. A flow rate of 0.2 ml/min was used. Starting composition was 32% B, which increased to 40% B at 1 min (held until 1.5 min) and then 45% B at 4 min. This was increased to 50% B at 5 min, 60% B at 8 min, 70% B at 11 min, and 80% B at 14 min (held until 16 min). At 16 min, the composition switched back to starting conditions (32% B) and was held for 4 min to re-equilibrate the column. Samples were analyzed in positive/negative switching ionization mode with top 5 data-dependent fragmentation utilizing a stepped collision energy of 25, 35, and 45 V. A resolution of 60,000 was utilized for full scan with a scan range of 200–1200 *m/z*. Data-dependent acquisition was acquired at a resolution of 15,000 and an isolation window of 1.5 *m/z*.

LC-MS data were analyzed by LipidSearch by ThermoFisher Scientific and the peak area was normalized to the area of deuterated internal standards from the Avanti Equisplash mix. Lipids were identified by MS^2^ fragmentation with the following parameters: precursor tolerance 5 ppm, product tolerance 8 ppm, m-score threshold 2.0, and product relative intensity threshold 0.1%. The higher-energy C-trap dissociation and labeled databases were used for identification. The identifications were generated individually for each sample and then aligned by grouping the samples.

### Fatty acid extraction and GC-MS analyses

For fatty acid extraction, a modified version of the microwave-assisted extraction method was utilized as previously described (25). Hundred milligrams of lung tissue was added to a microwave vessel [CEM Mars 6 microwave digestion system, equipped with a 24-vessel rotor and GlassChem vessel set (CEM Corporation, Matthews, NC)] with 8 ml of 4:1 (v/v) solution of ethyl acetate:methanol and 0.1% butylated hydroxytoluene. Fatty acids were extracted using the following microwave parameters: 55°C for 15 min with initial ramp of 2 min at 400W maximum power. Vessel contents were filtered using Whatman qualitative filter paper (Grade 597) into a test tube containing 3.5 ml HPLC-grade water. Samples were centrifuged at 2500 RPM for 6 min, and the top organic layer was transferred to a new tube and dried under nitrogen. Extracted oil was resuspended in 4:1 (v/v) dichloromethane:methanol with 0.1% butylated hydroxytoluene to bring each sample to 20 mg oil/ml. For the creation of fatty acid methyl esters (FAMEs), a modified methylation was conducted. Two milligrams of suspended oil (100 μl) was aliquoted from each sample, dried under nitrogen, and resuspended in toluene with 20 μg of internal standard (methyl 12-tridecenoate, U-35M, Nu-Chek Prep, Elysian, MN). Two milliliters of 0.5 N anhydrous potassium methoxide were added and samples were heated at 50°C for 10 min. Once cool, 3 ml of 5% methanolic HCl was added, and samples were heated at 80°C for 10 min. Once cool, 2 ml of water and 2 ml hexane were added, and the upper organic phase was removed and dried to obtain FAMEs. FAMEs were suspended in 1 ml isooctane to reach a concentration of 2 mg/ml and transferred to gas GC-MS vials with glass inserts. Samples were stored at −20°C until analysis.

For isolation of FAMEs, the PerkinElmer 680/600S GC– MS (Waltham, MA) in the electron impact mode was equipped with the Agilent Technologies (Santa Clara, CA) HP-88 column (100 m, 0.25 mm ID, 0.2 μM film thickness). The column temperature parameters were as follows: initial temperature at 80°C for 4 min; ramp 13.0°C/min to 175°C; hold 27 min; ramp 4.0°C/min to 215°C; hold 35 min. Helium was used as the carrier gas at a flow rate of 1 ml/min. Both a 30:1 split and a splitless injection were conducted for each sample at a 250°C injection temperature with a 1 μl sample volume. Samples were injected using two different injections to accurately quantify both lower concentration analytes and higher concentration analytes that were too concentrated on the splitless injection. The electron energy was 70ev and the MS data were recorded in full scan mode (mass range of m/z 70–400 amu). MS transfer line and ion source temperature were set at 180°C.

For identification of FAMEs, data analysis was conducted using MassLynx V4.1 SCN 714 (Waters Corporation, Milford, MA). Fatty acids were identified by retention time and EI mass fragmentation in comparison to that of our reference standard. Fatty acids were analyzed using extracted ion chromatograms of the respective quantitative ions. Fatty acids not included in our reference standard were identified according to elution order reported in the literature (26) and confirmed by the EI mass fragmentation. For quantification of FAMEs, we utilized a standard curve constructed from our reference and internal standard. The internal standard peak area and analyte peak area in the sample relative to that of standard curve was used to calculate each FAME concentration.

### Targeted liquid chromatography/tandem mass spectrometry for oxylipin analyses

PUFA-derived metabolites were analyzed from the left lung using a targeted mass spectrometry approach. Extensive details of the methods are as previously described (27). Briefly, lung samples were homogenized in cold methanol (−20°C) using a Qiagen Tissuelyser LT homogenizer (Qiagen, Germantown, Maryland) and the supernatant was extracted after centrifugation at 4°C and 14,000 rpm for 10 min. The supernatant was spiked with an internal standard solution (Cayman Chemical, Ann Arbor, MI) before drying to completion at 55°C under vacuum and reconstitution at 10% methanol by volume. The sample was then concentrated by elution through Strata-X 33-μm 30 mg/1 ml SPE columns (Phenomenex, Torrance, CA) using one volume of methyl formate and one volume of methanol which were both dried to completion under nitrogen. The resulting residue was reconstituted in 20 μl with ethanol and analyzed using two-dimensional reverse phase HPLC tandem mass spectrometry (liquid chromatography/tandem mass spectrometry or LC/MS/MS) equipped with an EclipsePlusC18 150 mm analytical HPLC column. All mass spectrometry and HPLC equipment was purchased from Agilent (Agilent technologies, Santa Clara, CA). All analytical reference standards were purchased form Cayman Chemical and all solvents were HPLC grade or higher. The parameters for LC/MS/MS along with their internal standards are provided in supplemental Table S1.

### Next generation RNA sequencing

RNA sequencing of the right lung was conducted as described (28). Briefly, RNA analytes were assayed for integrity, concentration, and fragment size. Total RNA-Seq library construction was performed from the RNA samples using the TruSeq Stranded RNA Sample Preparation Kit and bar-coded with individual tags following the manufacturer’s instructions (Illumina, Inc. San Diego, CA). An Agilent Bravo Automated Liquid Handling System was used to prepare libraries. Quality control was performed at every step and the libraries were quantified using a TapeStation system. Indexed libraries were prepared and run on the NovaSeq 6000 to generate an average of 35 million reads per sample. The raw Illumina sequence data were demultiplexed and converted to FASTSQ files, and adapter and low-quality sequences were quantified.

### Integrative omics

The data integration program xMWAS was used in R software v4.1.2 to integrate targeted oxylipin metabolomic data with lung RNA-seq transcriptomic data. xMWAS is programmed to use existing algorithms to generate an automated framework for differential network analysis for a maximum of four datasets from paired or unpaired study designs (23). Clustering and detection of communities within the datasets is established via the multilevel community detection method (29). Datasets were correlated by the eigenvector centrality measures with the absolute correlation threshold set to 0.4.

### ALI model

To induce lung injury, we exposed C57BL/6J mice, consuming either a lean control diet or HFD, to 1 ppm ozone for 3 h in individual wire mesh cages as previously described (30). Ozone was generated from oxygen (>90%), which was passed through a corona arc discharge chamber and metered into a Hazelton H-1000 chamber. A computer-operated feedback loop controlled the flow of ozone into the chamber based on concentration measurements made with a ThermoScientific Model 49i UV photometric ozone analyzer. Access to food and water was withheld for the exposure period and tissues were collected 24 h from the start of exposure. BALF was collected after sacrifice. Lung lobes were lavaged once with 0.5 ml and then again with 1 ml of saline. Cell differentials were acquired and oxylipins were measured as described above.

### Analyses

All data were analyzed for parametric distributions as previously described (18). A 2-way ANOVA with Šídák's multiple comparisons test was used for data from the glucose tolerance test. All other data sets were analyzed via unpaired two-tailed t-tests. All statistical analyses for all data, except transcriptomic data, were performed in Graphpad Prism version 9.3.0 where *P*-value < 0.05 was significant. One *ob/ob* mouse was removed from the study based on the Grubb’s method (Q = 1%) to identify outliers. For transcriptomic analyses, the quality for each fastq file was assessed using FASTQC to verify the absence of overabundant sequences, adapters, or poor quality scores (31). Bowtie 2 (32) was used to align the fastq files to the reference genome (*Mus musculus* UCSC MM10), resulting in SAM files. These files were then converted to BAM files and read into the subread featureCounts package for downstream analysis (33). The resulting feature count files were normalized and analyzed using the DESeq2 package within the R software v4.1.2. DESeq2 is a reliable approach that uses a negative binomial distribution with a linear model to adjust for false-positive significant results due to running statistical tests on a large number of genes (34). The output DESeq2 files contained Benjamini-Hochberg (BH)-adjusted *P*-values, log2 fold changes, and normalized raw counts for each gene. BH-adjusted *P*-value below 0.1 or a 10% false discovery rate was used for all downstream analysis. Each set of positive fold change or negative fold change genes with BH <0.1 was put through the Database for Annotation, Visualization, and Integrated Discovery (DAVID) Functional Annotation Tool v6.8 to generate the KEGG pathway clusters shared among the significant genes (35, 36). The DAVID tool helped determine which genes were enriched in any given KEGG pathway. Only KEGG pathways with BH p-adjusted <0.1 are reported here.

## Results

### The metabolic and pulmonary inflammatory profile of C57BL/6J mice consuming a HFD

**Figure 1:**
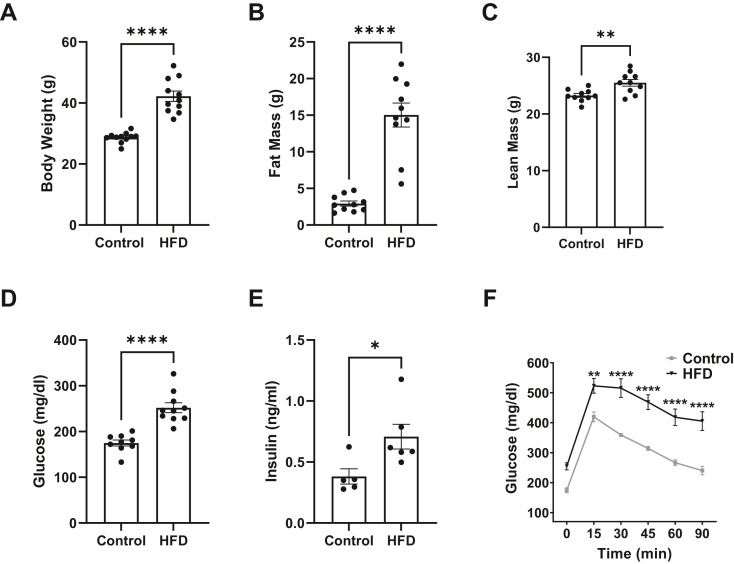
Metabolic profile of C57BL/6 mice consuming a high fat diet. (A) Body weights. (B) Fat mass and (C) lean mass obtained by Echo MRI. (D) Fasting glucose and (E) fasting insulin levels after a 5 h fast. (F) Glucose tolerance test completed after a 5 h fast by intraperitoneal injection of glucose. For all measurements, C57BL/6J male mice consumed a lean control or a high-fat diet (HFD) for 15 weeks. Metabolic characterization was conducted at 14 weeks of feeding (age 20–21 weeks). Data are mean ± SEM from 9-11 mice per group for (A) through (D), 4–5 mice per group for (E), and 7–9 mice per group for (F). ∗*P* < 0.05, ∗∗*P* <0.01, ∗∗∗∗*P* < 0.0001 from unpaired *t* test for (A) through (E) and 2-way ANOVA with Šídák's multiple comparisons test for F.

We first confirmed the metabolic state of the mice in response to the HFD. C57BL/6J mice showed significant elevation in body weight (Fig. 1A), which was driven by a pronounced increase in fat mass (Fig. 1B) and a small increase in lean mass (Fig. 1C) in response to the HFD relative to the lean controls. The mice were hyperglycemic (Fig. 1D), hyperinsulinemic (Fig. 1E), and displayed impaired glucose tolerance (Fig. 1F) with consumption of the HFD relative to controls. We characterized pulmonary inflammation of the mice in response to HFD by performing qRT-PCR on lung tissue for key proinflammatory cytokines and chemokines in the absence of external lung injury. We found no change in gene expression for any of the measured chemokines or cytokines for mice consuming a HFD relative to lean control (supplemental Fig. S1A–F). Additionally, there were no differences in total number of cells (supplemental Fig. S1G), the number of macrophages (supplemental Fig. S1H), or the number of neutrophils (supplemental Fig. S1I) in BALF for mice consuming a HFD relative to the lean control. The total protein in BALF was modestly higher in the HFD relative to the control with a *P*-value of 0.048 (supplemental Fig. S1J).

### The relative abundance of pulmonary triglycerides and phosphatidylethanolamines is dysregulated in response to a HFD

**Figure 2:**
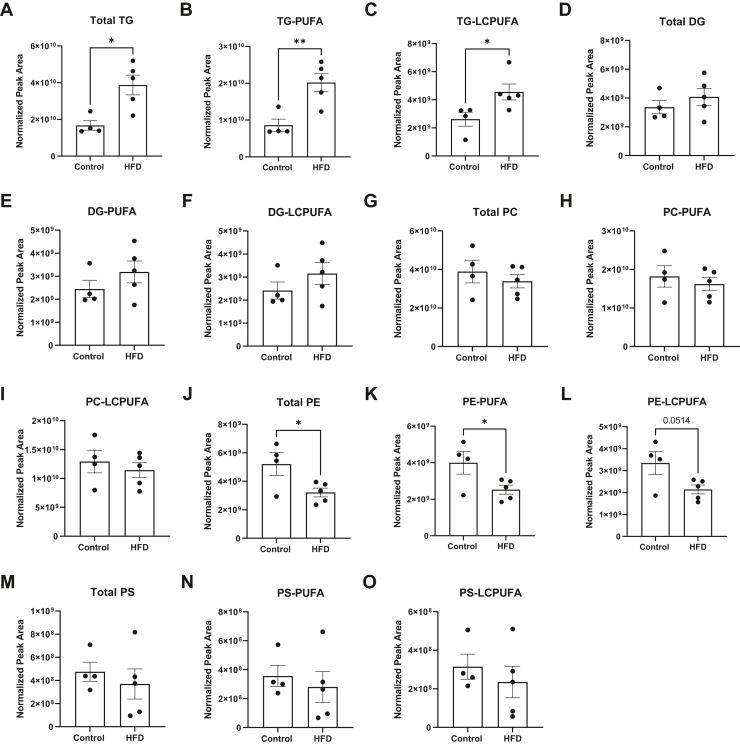
The relative abundance of pulmonary triglycerides (TG) and phosphatidylethanolamines are changed with a high fat diet. (A) Total TG, (B) PUFA-containing TG, and (C) long chain PUFA-containing (LCPUFA) TG. (D) Total diglycerides (DG), (E) PUFA-containing DG, and (F) LCPUFA-containing DG. (G) Total phosphatidylcholine (PC), (H) PUFA-containing PC, and (I) long chain PUFA-containing (LCPUFA) PC. (J) Total phosphatidylethanolamine (PE), (K) PUFA-containing PE, and (L) LCPUFA-containing PE. (M) Total phosphatidylserine (PS), (N) PUFA-containing PS, and (O) LCPUFA-containing PS. C57BL/6J male mice consumed a lean control or a high-fat diet (HFD) for 15 weeks. Isolated left lungs at age 21–22 weeks were used for untargeted mass spectrometry analysis. Data are mean ± SEM from 4-5 mice per group. ∗*P* < 0.05, ∗∗*P* <0.01 from unpaired 2-tailed *t* test.

Untargeted LC-MS was used to study the impact of a HFD on the broad pulmonary lipidome. These studies showed that mice consuming a HFD diet had a significant increase in the relative abundance of total triglyceride (TG) by ∼2-fold relative to the lean controls (Fig. 2A). PUFA-containing (Fig. 2B) and LC-PUFA-containing TGs (Fig. 2C) were increased by up to 2-fold with HFD relative to the lean controls. The HFD had no effect on total pulmonary diglycerides (Fig. 2D), PUFA-containing (Fig. 2E), or LC-PUFA-containing (Fig. 2F) diglycerides compared to the lean controls.

The relative abundance of pulmonary PUFA-containing phospholipids was also analyzed. Total phosphatidylcholines (Fig. 2G), PUFA-containing (Fig. 2H), and LC-PUFA-containing (Fig. 2I) phosphatidylcholines were not changed with the HFD. Total phosphatidylethanolamines (PE) were decreased with the HFD by 1.7-fold (Fig. 2J). PUFA-containing PEs (Fig. 2K) were also decreased with the HFD, and LC-PUFA-containing PEs showed a trend toward a reduction (*P*= 0.051) compared to the lean mice (Fig. 2L). There was no effect of the HFD on total phosphatidylserines (PSs) (Fig. 2M), PUFA-containing PS (Fig. 2N), or LC-PUFA-containing PS (Fig. 2O). In addition, there was no effect of the HFD, relative to the lean control, on total PI, PA, or PG-containing PUFAs (data not shown).

### Select pulmonary PUFA-derived oxylipins are increased with a HFD but are not increased due to an elevation in the total concentration of PUFAs

**Figure 3:**
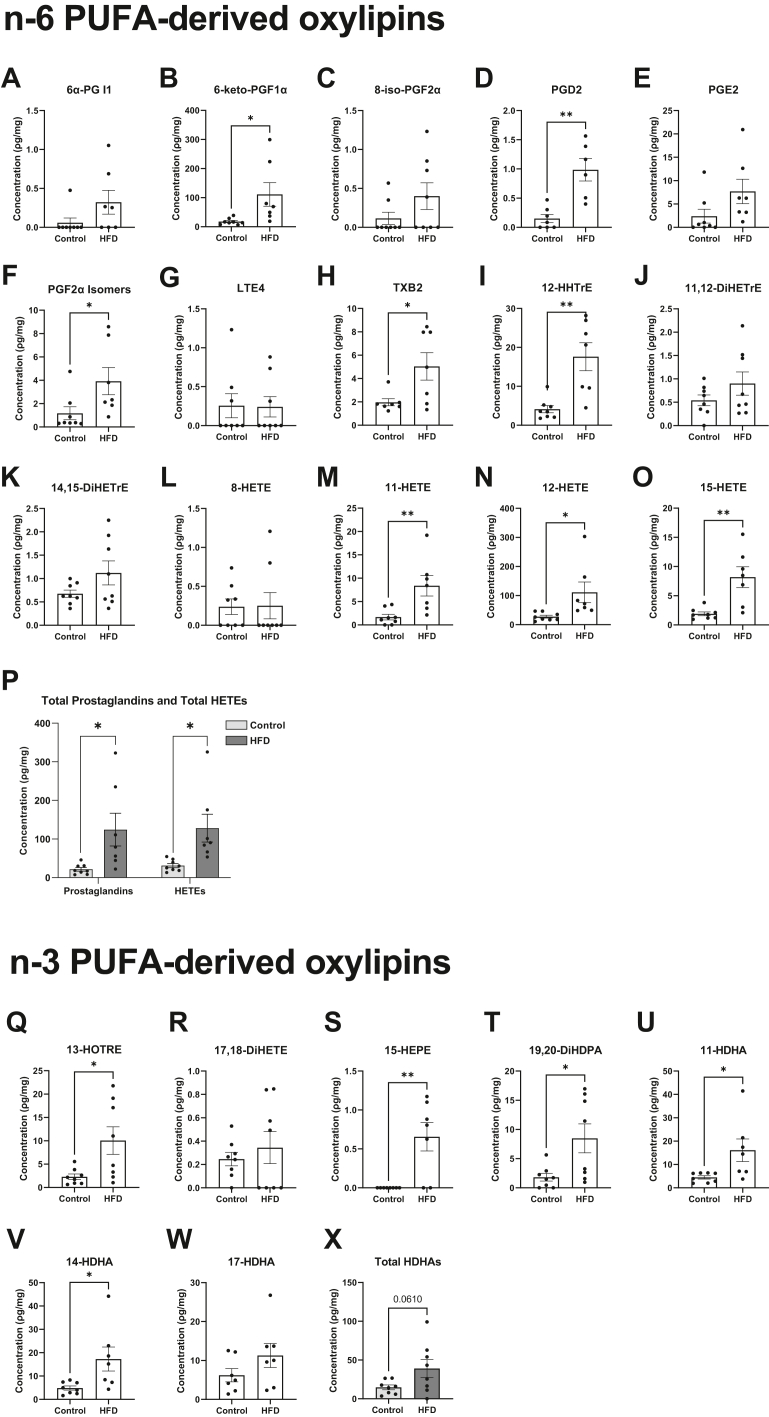
The concentration of select n-6 PUFA-derived and n-3 PUFA-derived oxylipins is increased with a high fat diet. (A) 6α-prostaglandin (PG) I1, (B) 6-keto-PGF1α, (C) 8-iso-PGF2α, (D) PGD2, (E) PGE2, (F) PGF2α isomers, (G) leukotriene E4 (LTE4), (H) thromboxane B2 (TXB2), (I) 12-hydroxyheptadecatrienoic acid (HHTrE), (J) 11,12-dihydroxyeicosatrienoic acid (11,12-DiHETrE), (K) 14,15-dihydroxyeicosatrienoic acid (14,15-DiHETrE), (L) 8-hydroxyeicosatetraenoic acid (8-HETE), (M) 11-hydroxyeicosatetraenoic acid (11-HETE), (N) 12-hydroxyeicosatetraenoic acid (12-HETE), (O) 15-hydroxyeicosatetraenoic acid (15-HETE), (P) total prostaglandins and total HETEs, (Q) 13-hydroxyoctadecatrienoic acid (13-HOTRE), (R) 17,18-hydroxyeicosatetraenoic acid (17,18-DiHETE), (S) 15-hydroxyeicosapentaenoic acid (15-HEPE), (T) 19,20-dihydroxydocosapentaenoic acid (19,20-DiHDPA), (U) 11-hydroxydocosahexaenoic acid (11-HDHA), (V) 14-hydroxydocosahexaenoic acid (14-HDHA), (W) 17-hydroxydocosahexaenoic acid (17-HDHA), and (X) total HDHAs. C57BL/6J male mice consumed a lean control or a high-fat diet (HFD) for 15 weeks. Isolated left lungs at 21–22 weeks of age were used for targeted mass spectrometry analysis. Data are mean ± SEM from 7-8 mice per group. ∗*P* < 0.05, ∗∗*P* <0.01 from unpaired *t* test.

We next analyzed how a HFD controlled the concentration of metabolites synthesized from n-6 and n-3 LC-PUFAs that are central in inflammation initiation and resolution (Fig. 3A–X). Select AA-derived oxylipins were significantly elevated in response to HFD relative to lean control. 6-keto-PGF1α (Fig. 3B), PGD2 (Fig. 3D), and PGF2α Isomers (Fig. 3F), respectively, had 6-fold, 6.5-fold, and 4-fold increases, with the HFD relative to control. TXB2 (Fig. 3H) and 12-HHTrE (Fig. 3I) increased by 4-fold with the HFD relative to control. Additionally, 11-HETE (Fig. 3M) had a 5-fold increase, whereas 12-HETE (Fig. 3N) and 15-HETE (Fig. 3O) had a 4-fold increase in response to the HFD relative to control. Overall, total prostaglandins and HETEs were elevated with the HFD relative to the lean control (Fig. 3P). Linoleic acid-derived oxylipins were not changed for HFD compared to control (data not shown).

N-3 PUFA-derived oxylipins were also increased with the HFD relative to the lean control (Fig. 3Q–W). Notably, 13-HOTRE (Fig. 3Q) and 15-HEPE (Fig. 3S) increased by 4-fold and 6-fold in response to the HFD relative to control, respectively. 19,20-DiHDPA (Fig. 3T), 11-HDHA (Fig. 3U), and 14-HDHA (Fig. 3V) were all increased by at least 4-fold in response to the HFD relative to control. Overall, the total concentration of HDHAs showed a trend toward an increase (*P* = 0.06) with the HFD compared to the lean control (Fig. 3X).

We next determined if the increase in n-6 and n-3 PUFA-derived oxylipins was driven by an increase in the pulmonary concentration of the parent fatty acids for oxylipins. We analyzed all of the major pulmonary fatty acids (supplemental Fig. S2A–H). The key fatty acids of interest, AA, (supplemental Fig. S2D), EPA (supplemental Fig. S2G), and DHA (supplemental Fig. S2H), did not change with the HFD relative to the lean control. DPAn-3 levels were increased with the HFD compared to the lean mice (supplemental Fig. S2F). This indicated that the increase in oxylipin levels was not driven by an increase in the concentration of AA, EPA, or DHA.

### Genetic model of obesity mimics the signature of PUFA-derived oxylipins driven by a HFD in the absence of external lung injury

The next question was whether the effects of the HFD diet were specific to this model or also applicable with another model of obesity. Therefore, the concentration of pulmonary PUFA-derived oxylipins were analyzed from *ob/ob* mice, a genetic model of obesity. The *ob/ob* mice relative to lean controls showed a significant difference in body weight (supplemental Fig. S3A), which was associated with an increased in fat mass (supplemental Fig. S3B) and a reduction in lean mass (supplemental Fig. S3C). The *ob/ob* mice were not hyperglycemic (supplemental Fig. S3D) but were hyperinsulinemic (supplemental Fig. S3E) and had impaired glucose tolerance (supplemental Fig. S3F) relative to controls.

**Figure 4:**
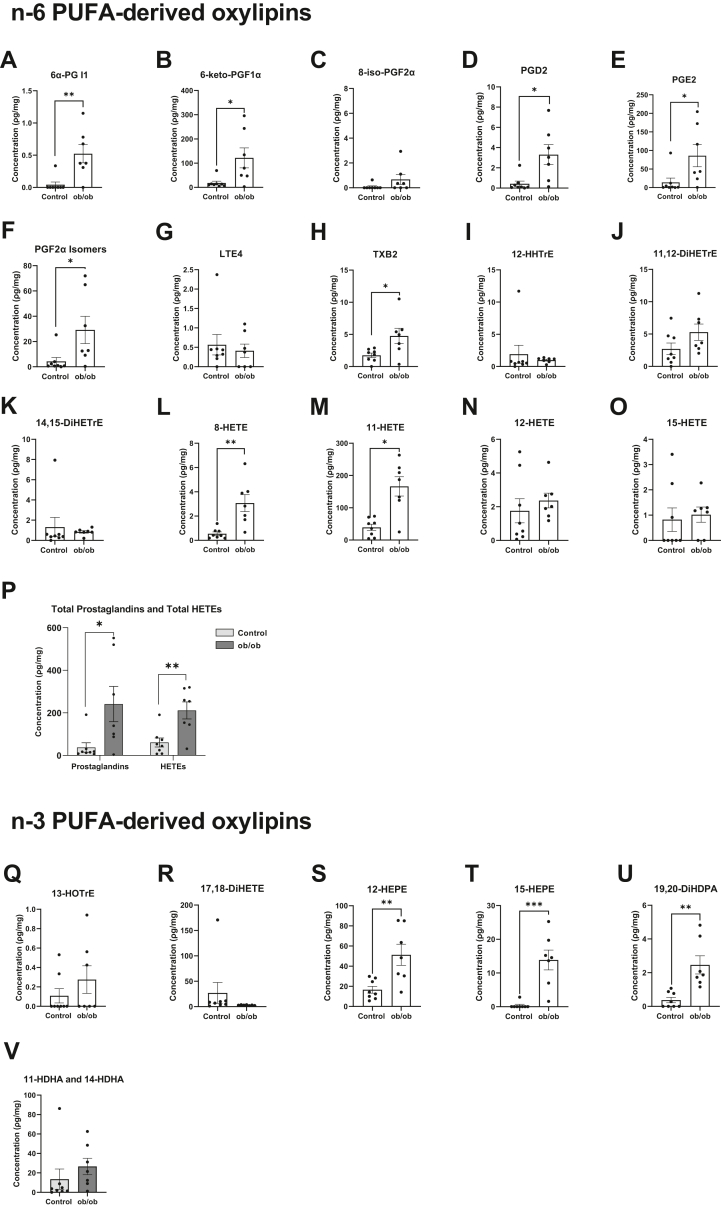
The concentration of select n-6 PUFA-derived and n-3 PUFA-derived oxylipins in a genetic model of obesity. (A) 6α-prostaglandin (PG) I1, (B) 6-keto-PGF1α, (C) 8-iso-PGF2α, (D) PGD2, (E) PGE2, (F) PGF2α isomers, (G) leukotriene E4 (LTE4), (H) thromboxane B2 (TXB2), (I) 12-hydroxyheptadecatrienoic acid (HHTrE), (J) 11,12-dihydroxyeicosatrienoic acid (11,12-DiHETrE), (K) 14,15-dihydroxyeicosatrienoic acid (14,15-DiHETrE), (L) 8-hydroxyeicosatetraenoic acid (8-HETE), (M) 11-hydroxyeicosatetraenoic acid (11-HETE), (N) 12-hydroxyeicosatetraenoic acid (12-HETE), (O) 15-hydroxyeicosatetraenoic acid (15-HETE), (P) total prostaglandins and total HETEs, (Q) 13-hydroxyoctadecatrienoic acid (13-HOTrE), (R) 17,18-hydroxyeicosatetraenoic acid (17,18-DiHETE), (S) 12-hydroxyeicosapentaenoic acid (12-HEPE), (T) 15-hydroxyeicosapentaenoic acid (15-HEPE), (U) 19,20-dihydroxydocosapentaenoic acid (19,20-DiHDPA), (V) total 11-hydroxydocosahexaenoic acid (11-HDHA) and 14-hydroxydocosahexaenoic acid (14-HDHA). Genetically obese (*ob/ob*) male mice and lean control male mice were purchased from Jackson Laboratory at 7 weeks of age and were fed normal chow for 3 weeks. Isolated left lungs at age 10–11 weeks were used for targeted mass spectrometry analysis, where the body weights were matched with the body weights of mice fed a high fat diet. Data are mean ± SEM from 7-8 mice per group. ∗*P* < 0.05, ∗∗*P* <0.01, ∗∗∗*P* < 0.001 from unpaired *t* test.

We analyzed pulmonary AA-derived oxylipins with *ob/ob* mice relative to control mice (Fig. 4A–O) and found similar increases in select oxylipins as we did with the HFD model. 6α-PGI1 (Fig. 4A), 6-keto-PGF1α (Fig. 4B), PGD2 (Fig. 4D), PGE2 (Fig. 4E), and PGF2α isomers (Fig. 4F) were respectively increased by 12-fold, 6.7-fold, 7.8-fold, 6-fold, and 6.7-fold with *ob/ob* mice relative to control mice. TXB2 (Fig. 4H), 8-HETE (Fig. 4L), 11-HETE (Fig. 4M) were respectively increased by 2.7-fold, 5.6-fold, and 4.2-fold with *ob/ob* mice compared to lean mice. Overall, total prostaglandins and HETEs were elevated with the genetic model of obesity relative to controls by 6.5-fold and 3.5-fold, respectively (Fig. 4P).

Some n-3 PUFA-derived oxylipins were also increased with *ob/ob* mice relative to the lean controls (Fig. 4Q–U). Notably, 12-HEPE (Fig. 4S), 15-HEPE (Fig. 4T), and 19,20-DiHDPA (Fig. 4U) were respectively increased by 3-fold, 39-fold, and 6.6-fold with *ob/ob* mice compared to controls. In contrast, there was no change in total HDHAs with the genetic model of obesity relative to controls (Fig. 4V).

### Pulmonary transcriptomic analyses via next generation RNA sequencing reveal complex and unique pathways by which the HFD may dysregulate immunity and lipid metabolism

**Figure 5:**
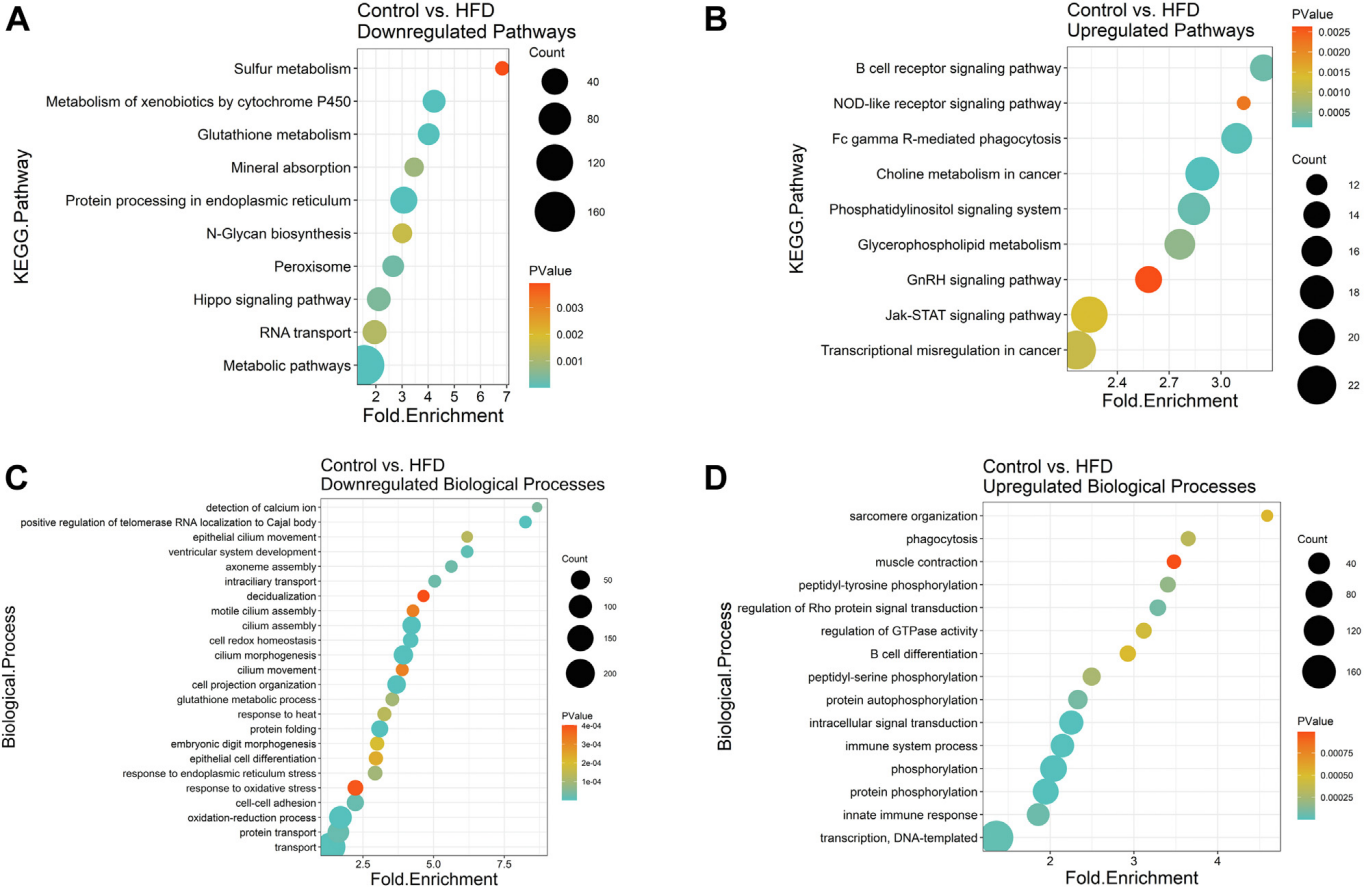
Transcriptome analyses via RNA-sequencing reveal an upregulation of immune-related and lipid-metabolism–related pathways and biological processes in response to high fat diet. KEGG (Kyoto Encyclopedia of Genes and Genomes) pathways and gene ontology biological processes are depicted in the following dot plots: (A) downregulated KEGG pathways, (B) upregulated KEGG pathways, (C) downregulated biological processes, and (D) upregulated biological processes. C57BL/6J male mice consumed a control diet or an experimental high-fat diet (HFD) for 15 weeks. N = 7–8 mice per group. All depicted pathways and biological processes are significant based on differential gene expression (DeSeq) analysis with Benjamini-Hochberg p-adjusted < 0.1.

We conducted RNAseq analyses of the lungs to investigate how the HFD manipulates the underlying transcriptome, particularly as it relates to PUFA metabolism and immunity. KEGG pathway analyses showed that the HFD downregulated (Fig. 5A) and upregulated several pathways (Fig. 5B). Interestingly, B cell receptor signaling showed the strongest upregulation in addition to glycerophospholipid metabolism with the HFD. The genes included in each of the downregulated and upregulated KEGG pathways are presented in supplemental Tables S2 and S3, respectively. The HFD also decreased (Fig. 5C) and increased (Fig. 5D) many biological processes. These notably included B cell differentiation in addition to various immune system processes. The genes included in each of the downregulated and upregulated biological processes are presented in supplemental Tables S4 and S5, respectively. The top 15 genes for select pathways/processes were used to make heatmaps for B-cell differentiation (supplemental Fig. S4A), B-cell receptor signaling (supplemental Fig. S4B), cell redox homeostasis (supplemental Fig. S4C), glycerophospholipid metabolism (supplemental Fig. S4D), immune system (supplemental Fig. S4E), innate immune (supplemental Fig. S4F), peroxisome (supplemental Fig. S4G), phagocytosis (supplemental Fig. S4H), phosphatidylinositol signaling (supplemental Fig. S4I), and response to oxidative stress (supplemental Fig. S4J).

### Integration of lipidomic and transcriptomics reveals novel gene-oxylipin networks driven by a HFD

Integrative omics were performed for the targeted lipidomic and transcriptomic data to see if there was any meaningful relationship across oxylipins and genes in a given pathway. Overall, we found several distinct oxylipin-gene networks for the HFD relative to lean control (data not shown). We decided to focus on networks for genes in the glycerophospholipid metabolism and B cell receptor signaling pathways, which were some of the most upregulated pathways. Furthermore, the rationale for focusing on glycerophospholipid metabolism is that it is central to oxylipin metabolism; in addition, the role of pulmonary B cell receptor signaling in obesity is completely unknown. The analysis resulted in network threshold values, presented in supplemental Table S6, between the gene and the oxylipins which translated to either a positive or negative correlation.

**Figure 6:**
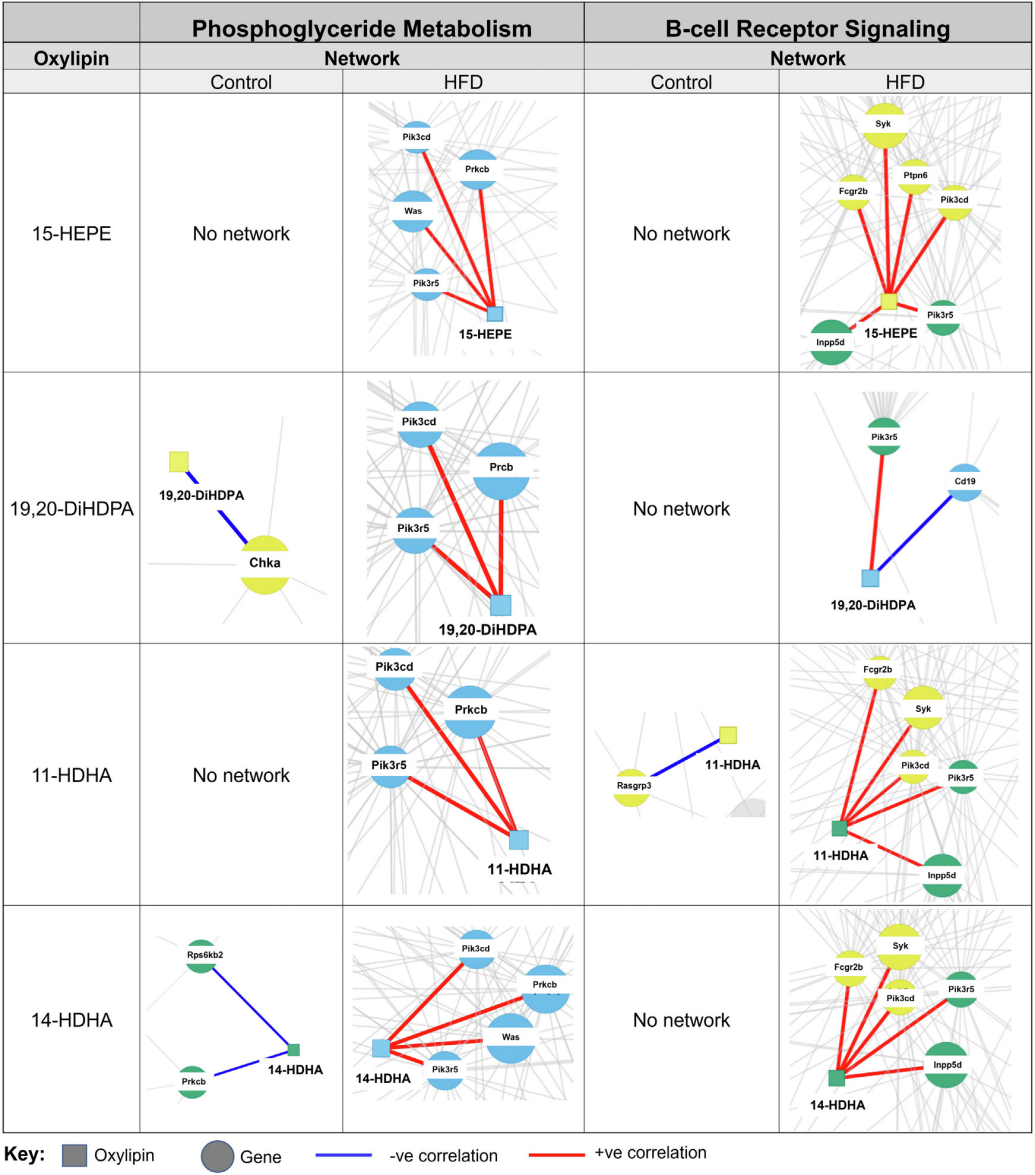
Integrative omics of transcriptomic and metabolomic analyses shows different network interactions for key n-3 PUFA-derived oxylipins with a high fat diet. Differential network analysis for selective n-3 PUFA-derived oxylipins (15-HEPE, 19,20-DiHDPA, 11-HDHA, and 14-HDHA) was conducted for genes present in the Phosphoglyceride metabolism and B-cell receptor signaling pathways using xMWAS, a software program for data integration. Squares represent metabolites and circles represent genes. Clustering of data resulted in distinct communities which are represented by different colors for genes and metabolites. Blue lines represent a negative correlation and red lines represent a positive correlation between a given oxylipin and gene.

For glycerophospholipid metabolism (left panel, Fig. 6), there was clearly more integration between 15-HEPE, 19,20-DiHDPA, 11-HDHA, and 14-HDHA with the HFD compared to lean mice. Notably, these four oxylipins were positively correlated with the genes *Pik3cd*, *Pik3r5*, and *Prkcb* with the HFD. Additionally, 15-HEPE and 14-HDHA also positively correlated with the gene *Was* in response to the HFD. There was no network with any genes from glycerophospholipid metabolism for 15-HEPE or 11-HDHA with the control diet. For the control, 19,20-DiHDPA negatively correlated with the gene *Chka* and 14-HDHA negatively correlated with the genes *Rps6kb2* and *Prkcb*.

Similar to the glycerophospholipid analyses, it was clear that more networks exist for the HFD compared to the lean mice when analyzing the data for B cell receptor signaling (right panel, Fig. 6). Notably, 15-HEPE, 11-HDHA, and 14-HDHA positively correlated with the genes *Fcgr2b*, *Syk*, *Pik3cd*, *Pik3r5*, *Inpp5d* in response to the HFD. Additionally, 15-HEPE also positively correlated with *Ptpn6* with the HFD. 19,20-DiHDPA positively correlated with the gene *Pik3r5* and negatively correlated with the gene *Cd19* in response to the HFD. There was no network for 15-HEPE, 19,20-DiHDPA, and 14-HDHA with the control diet. 11-HDHA was negatively correlated with the gene *Rasgrp3* in response to the control diet.

### The pulmonary inflammatory and oxylipin signature are generally dysregulated with a HFD upon ALI

**Figure 7:**
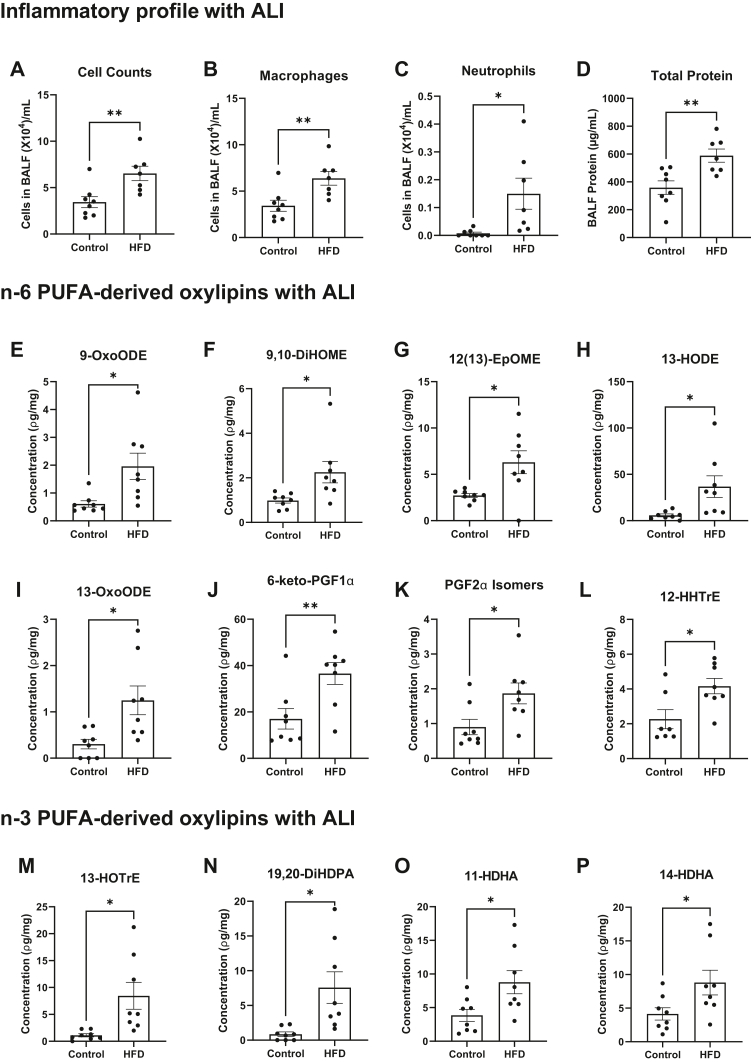
Lung inflammation is exacerbated and the concentration of select oxylipins is increased with a high fat diet upon ALI. (A) Cell counts, (B) number of macrophages, (C) number of neutrophils, and (D) total protein in bronchoalveolar fluid (BALF). (E) 9-hydroxyoctadecenoic acid (9-HODE), (F) 9,10-dihydroxyoctadecenoic acid (9,10-DiHOME), (G) 12,13-epoxyoctadecenoic acid (12,13-EpOME), (H) 13-hydroxyoctadecenoic acid (13-HODE), (I) 13-oxooctadecenoic acid (13-OxoODE), (J) 6-keto-PGF1α, (K) PGF2α isomers, (L) 12-hydroxyheptadecatrienoic acid (12-HHTrE), (M) 13-hydroxyoctadecatrienoic acid (13-HOTRE), (N) 19,20-dihydroxydocosapentaenoic acid (19,20-DiHDPA), (O) 11-hydroxydocosahexaenoic acid (11-HDHA), (P) 14-hydroxydocosahexaenoic acid (14-HDHA). C57BL/6J male mice consumed a lean control or a high-fat diet (HFD) for 15 weeks and were exposed to 1 ppm O3 for 3 h and were euthanized 24 h after exposure. Isolated left lungs at 21–22 weeks of age were used for targeted mass spectrometry analysis. Data are mean ± SEM from 7-8 mice per group. ∗*P* < 0.05 and ∗∗*P* <0.01 from unpaired *t* test.

Finally, we investigated if PUFA-derived oxylipins and inflammatory markers were also dysregulated upon ALI. Exposure of mice to ozone caused a 2-fold increase in cell counts (Fig. 7A), a 2-fold increase in macrophages (Fig. 7B), a 19-fold increase in neutrophils (Fig. 7C), and a 1.6-fold increase in total protein (Fig. 7D) in BALF of mice on a HFD compared to lean controls. In terms of the oxylipin pulmonary profile, several n-6 PUFA-derived oxylipins were elevated with the HFD upon exposure to ozone, which included 9-OxoODE (Figs. 7E), 9,10-DiHOME (Fig. 7F), 12(13)-EpOME (Fig. 7G), 13-HODE (Fig. 7H), 13-OxoODE (Fig. 7I), 6-keto-PGF1α (Fig. 7J), PGF2a isomers, (Fig. 7K), and 12-HHTrE (Fig. 7L). Some n-3 PUFA-derived oxylipins were also elevated with the HFD, which were 13-HoTrE (Figs. 7M), 19,20-DiHDPA (Fig. 7N), 11-HDHA (Fig. 7O), and 14-HDHA (Fig. 7P). Thus, these results showed that the concentration of pulmonary oxylipins is dysregulated with a HFD upon ALI.

## Discussion

Obesity is associated with poor outcomes for differing pulmonary diseases, including, but not limited to asthma, acute respiratory distress syndrome, and COPD. Obesity also increases the risk for pulmonary infections, including influenza and SARS-CoV-2 (9, 37, 38). Thus, there is an urgent need to investigate how obesity increases the risk for differing pulmonary diseases and infections. Herein, we focused on how HFD-induced obesity may dysregulate a key aspect of pulmonary lipid metabolism related to inflammation initiation and resolution.

One key finding was that obese mice had increased triglycerides in pulmonary tissue. Excess triglycerides are important as they impair immunological pathways in metabolic organs (39). The data are consistent with a previous study that showed obesity, driven by a high fat or high sugar diet, promoted dysregulation in the pulmonary triglyceride and phosphatidylethanolamine pools although in this study, some lipid species were elevated while others were decreased (40). The data were also in agreement with a human study that showed adipose tissue accumulates in the airway walls with increasing body-mass-index (41). Interestingly, this study showed that increased adipose tissue accumulation in airway walls correlated with an increased number of neutrophils (41). Thus, it is conceivable that accumulation of triglycerides in the lungs may be driven by enrichment of adipose tissue in the airway walls, which warrants further investigation. Future studies will need to investigate if increased accumulation of triglycerides in the airways or within pulmonary tissue is leading to increased accumulation of fatty acids into differing lipid pools (i.e., neutral lipids, polar lipids, surfactant). A good starting point would be to investigate which cell types in pulmonary tissue are accumulating triglycerides.

The targeted lipidomic analyses showed that oxylipins synthesized from AA were increased with the HFD, suggesting initiation of inflammation prior to external lung injury. A novel finding is the increase in AA-derived oxylipins is occurring in the absence of any increase in markers of inflammation in the BALF. A critical next step will be to determine the cellular source of the increase in AA-derived oxylipins in obese mice. We also found that n-6 PUFA-derived oxylipins were increased upon ALI to mice fed HFD with a marked increase of neutrophils and macrophages in BALF, consistent with the notion that obesity promotes lung injury and inflammation with ozone exposure (42, 43). Previous work shows that lungs from obese mice, in the absence of any induced injury, are not inflamed and show no damage by histopathology (8). However, the increase in select oxylipins suggest that inflammation may be initiated with obesity prior to overt lung inflammation, and perhaps this is mechanistically linked to the increase in triglycerides.

The increase in pulmonary n-3 PUFA-derived oxylipins was unexpected based on our previous work. Obesity is generally associated with a reduction in oxylipins generated from n-3 PUFAs. For instance, EPA- or DHA-derived metabolites were decreased in white adipose tissue, liver, spleen, and bone marrow of obese male mice (18, 20, 21). Thus, our data raise the question of whether the increase in some of these oxylipins in the lungs reflects a potential redistribution from other metabolic tissues. Another possibility is that the increase in oxylipins reflects an environment that is becoming inflamed. Our data were not in agreement with a study that showed no change in pulmonary n-3 PUFA-derived oxylipins when comparing mice consuming a Western diet relative with lean controls (44). This could be due to the differences in diet. This previous study did show that a Western diet decreased the expression of SPM receptors, which likely impaired the inflammatory response to silver nanoparticles (44).

The parent fatty acids for oxylipins were generally not increased with the C57BL/6J mice fed HFD. These results reveal that the increase in oxylipins is likely not due to an increase in the parent fatty acids, suggesting potential changes in the abundance or activity of PUFA-metabolizing enzymes. In support of this possibility, recent work show that dysregulation in oxylipin signatures of the n-3 PUFA family in humans with obesity was not driven by a change in the concentration of EPA or DHA (22). A key study demonstrated that the impairment in n-3 PUFA-derived oxylipin levels in obesity was due to a defect, at least in the case of leukocyte SPM levels, in 5- and 12/15-lipoxygenase phosphorylation or subcellular localization (22).

The transcriptomic studies revealed that numerous biological pathways and processes were dysregulated in response to the HFD. We specifically focused on two key pathways, which were related to B cells and phospholipid metabolism. The increase in B cell differentiation and signaling was of strong interest for several reasons. One, select B cell subsets are drivers of inflammation and B2 cells infiltrate white adipose tissue and promote inflammation through a leukotriene B4-mediated mechanism (16, 45). Second, 14-HDHA and 17-HDHA control varying aspects of B cell differentiation such as increasing the abundance of CD138^+^ antibody secreting cells (20, 46, 47). Thus, it is possible that the increase in pulmonary 14-HDHA with the HFD may be influencing B cell enrichment and/or differentiation as it relates to the lungs. The role of B cells in pulmonary inflammation in the context of obesity is completely unknown (48). The computational analyses showed that 14-HDHA was positively associated with several genes including *Fcgr2b*, which is of interest as Fc receptors have a role in controlling autoreactive B cells and production of autoantibodies (49). Therefore, future studies will need to examine how 14-HDHA may be controlling antibody production in the lungs. It will be of interest to investigate how a HFD controls differing B cell subsets using flow cytometry in addition to investigating the impact of a HFD on other immune cell populations that are relevant for initiating or resolving inflammation in the lungs.

The transcriptomic data also revealed significant changes in numerous genes related to phospholipid metabolism. For instance, *Pla2g4b* and *Pla2g4F* were increased with the HFD. These genes encode for phospholipase A2, which is intriguing as the upregulation of these genes may reflect increased cleavage of PUFA-containing phospholipids and thereby an increase in PUFA-derived oxylipins. Perhaps the increased gene expression of phospholipases is driven by systemic metabolic dysregulation in response to obesity. For instance, there is some evidence to show that leptin stimulates respiratory expression of cytosolic phospholipase A2a in addition to cyclooxygenase-2 (50). We also found increased networks between specific oxylipins and genes related to PIK3s, which coordinate a range of functions related to inflammation and immunity. The molecular details remain to be established by which specific oxylipins control the expression of differing genes or vice-versa.

There are some limitations to this study. First, we did not conduct studies with female mice as there is established sex differences in oxylipin profiles and inflammation, particularly in the context of obesity (21). Second, we did not establish the underlying mechanisms by which obesity drives an increase in pulmonary PUFA-derived oxylipins. The *ob/ob* model generally recapitulated the results from the HFD model. This suggests that the state of obesity, and not a high fat diet, is driving the dysregulation of pulmonary oxylipins. This opens the door to the possibility that metabolic factors that are common to both models of obesity may have a role in controlling the pulmonary oxylipin and inflammatory response, which is an area for future investigation. Finally, the transcriptomic data will require further investigation. As an example, we observed some upregulation in pathways related to cancer. This is relevant as murine obesity promotes cancer progression and metastasis (51); therefore, obesity may be targeting pathways that are of interest to lung cancer development.

In summary, these studies reveal that obesity may be priming the lungs for exacerbated inflammation prior to external lung injury. In particular, PUFA metabolism is dysregulated at a molecular level accompanied by strong changes in a variety of biological process and pathways. Some of these pathways, including a unique elevation in B cell receptor signaling, are potentially linked to changes in oxylipin signaling. Overall, the work has strong implications for understanding why obesity drives a poor response upon infectious and inflammatory challenges. This is highly relevant given that obesity is a risk factor for differing pulmonary autoimmune diseases and infections including SARS-CoV-2, which is driving the current COVID-19 pandemic.

## Data availability

All data will be made available upon reasonable request. Please direct questions to the corresponding author, Dr Saame Raza Shaikh: shaikhsa@email.unc.edu. Untargeted lipidomic data are deposited into the metabolomic workbench DataTrack ID 3104 and study ID ST002093. Targeted lipidomic data are deposited in MetaboLights with the unique identifier, MTBLS5224, www.ebi.ac.uk/metabolights/MTBLS5224 (52). Transcriptomic data are deposited in NCBI's Gene Expression Omnibus (53) and are accessible through GEO Series accession number GSE194403 (https://www.ncbi.nlm.nih.gov/geo/query/acc.cgi?acc=GSE194403).

## *Supplemetal data*

This article contains supplemental data.

## Conflicts of interest

S. R. S. has received industry support on projects and conferences related to n-3 fatty acids from Metagenics Inc., and the Wiley Companies.

## References

[1] Ogden C.L., Carroll M.D., Curtin L.R., McDowell M.A., Tabak C.J., Flegal K.M. (2006). Prevalence of overweight and obesity in the United States, 1999-2004. JAMA.

[2] Dutton G.R., Kim Y., Jacobs D.R., Li X., Loria C.M., Reis J.P. (2016). 25-year weight gain in a racially balanced sample of U.S. adults: the CARDIA study. Obesity.

[3] Ather J.L., Van Der Vliet K.E., Mank M.M., Reed L.F., Dixon A.E., Poynter M.E. (2021). Obese adipose tissue modulates proinflammatory responses of mouse airway epithelial cells. Am. J. Physiol. Regul. Integr. Comp. Physiol..

[4] Plataki M., Fan L., Sanchez E., Huang Z., Torres L.K., Imamura M. (2019). Fatty acid synthase downregulation contributes to acute lung injury in murine diet-induced obesity. JCI Insight.

[5] Alqahtani S., Kobos L.M., Xia L., Ferreira C., Franco J., Du X. (2020). Exacerbation of nanoparticle-induced acute pulmonary inflammation in a mouse model of metabolic syndrome. Front. Immunol..

[6] Hsu Y.-E., Chen S.-C., Geng J.-H., Wu D.-W., Wu P.-Y., Huang J.-C. (2021). Obesity-related indices are associated with longitudinal changes in lung function: a large taiwanese population follow-up study. Nutrients.

[7] de Boer G.M., Tramper-Stranders G.A., Houweling L., van Zelst C.M., Pouw N., Verhoeven G.T. (2021). Adult but not childhood onset asthma is associated with the metabolic syndrome, independent from body mass index. Respir. Med..

[8] Milner J.J., Rebeles J., Dhungana S., Stewart D.A., Sumner S.C.J., Meyers M.H. (2015). Obesity increases mortality and modulates the lung metabolome during pandemic H1N1 influenza virus infection in mice. J. Immunol..

[9] Popkin B.M., Du S., Green W.D., Beck M.A., Algaith T., Herbst C.H. (2020). Individuals with obesity and COVID-19: a global perspective on the epidemiology and biological relationships. Obes. Rev..

[10] Serhan C.N., Levy B.D. (2018). Resolvins in inflammation: emergence of the pro-resolving superfamily of mediators. J. Clin. Invest..

[11] Karp C.L., Flick L.M., Park K.W., Softic S., Greer T.M., Keledjian R. (2004). Defective lipoxin-mediated anti-inflammatory activity in the cystic fibrosis airway. Nat. Immunol..

[12] Posso S.V., Quesnot N., Moraes J.A., Brito-Gitirana L., Kennedy-Feitosa E., Barroso M.V. (2018). AT-RVD1 repairs mouse lung after cigarette smoke-induced emphysema via downregulation of oxidative stress by NRF2/KEAP1 pathway. Int. Immunopharmacol..

[13] Miyata J., Arita M. (2015). Role of omega-3 fatty acids and their metabolites in asthma and allergic diseases. Allergol. Int..

[14] Cagnina R.E., Duvall M.G., Nijmeh J., Levy B.D. (2022). Specialized pro-resolving mediators in respiratory diseases. Curr. Opin. Clin. Nutr. Metab. Care.

[15] Li P., Oh D.Y., Bandyopadhyay G., Lagakos W.S., Talukdar S., Osborn O. (2015). LTB4 promotes insulin resistance in obese mice by acting on macrophages, hepatocytes and myocytes. Nat. Med..

[16] Ying W., Wollam J., Ofrecio J.M., Bandyopadhyay G., El Ouarrat D., Lee Y.S. (2017). Adipose tissue B2 cells promote insulin resistance through leukotriene LTB4/LTB4R1 signaling. J. Clin. Invest..

[17] Neuhofer A., Zeyda M., Mascher D., Itariu B.K., Murano I., Leitner L. (2013). Impaired local production of proresolving lipid mediators in obesity and 17-HDHA as a potential treatment for obesity-associated inflammation. Diabetes.

[18] Pal A., Al-Shaer A.E., Guesdon W., Torres M.J., Armstrong M., Quinn K. (2020). Resolvin E1 derived from eicosapentaenoic acid prevents hyperinsulinemia and hyperglycemia in a host genetic manner. FASEB J..

[19] Clària J., Dalli J., Yacoubian S., Gao F., Serhan C.N. (2012). Resolvin D1 and resolvin D2 govern local inflammatory tone in obese fat. J. Immunol..

[20] Kosaraju R., Guesdon W., Crouch M.J., Teague H.L., Sullivan E.M., Karlsson E.A. (2017). B cell activity is impaired in human and mouse obesity and is responsive to an essential fatty acid upon murine influenza infection. J. Immunol..

[21] Crouch M.J., Kosaraju R., Guesdon W., Armstrong M., Reisdorph N., Jain R. (2019). Frontline Science: a reduction in DHA-derived mediators in male obesity contributes toward defects in select B cell subsets and circulating antibody. J. Leukoc. Biol..

[22] López-Vicario C., Titos E., Walker M.E., Alcaraz-Quiles J., Casulleras M., Durán-Güell M. (2019). Leukocytes from obese individuals exhibit an impaired SPM signature. FASEB J..

[23] Uppal K., Ma C., Go Y.-M., Jones D.P. (2018). xMWAS: a data-driven integration and differential network analysis tool. Bioinformatics.

[24] Kilburg-Basnyat B., Reece S.W., Crouch M.J., Luo B., Boone A.D., Yaeger M. (2018). Specialized pro-resolving lipid mediators regulate ozone-induced pulmonary and systemic inflammation. Toxicol. Sci..

[25] Bronkema S.M., Rowntree J.E., Jain R., Schweihofer J.P., Bitler C.A., Fenton J.I. (2019). A nutritional survey of commercially available grass-finished beef. Meat and Muscle Biol..

[26] Kramer J.K.G., Hernandez M., Cruz-Hernandez C., Kraft J., Dugan M.E.R. (2008). Combining results of two GC separations partly achieves determination of all cis and trans 16:1, 18:1, 18:2 and 18:3 except CLA isomers of milk fat as demonstrated using Ag-ion SPE fractionation. Lipids.

[27] Armstrong M., Manke J., Nkrumah-Elie Y., Shaikh S.R., Reisdorph N. (2020). Improved quantification of lipid mediators in plasma and tissues by liquid chromatography tandem mass spectrometry demonstrates mouse strain specific differences. Prostaglandins Other Lipid Mediat..

[28] Al-Shaer A.E., Pal A., Shaikh S.R. (2021). Resolvin E1-ChemR23 axis regulates the hepatic metabolic and inflammatory transcriptional landscape in obesity at the whole genome and exon level. Front. Nutr..

[29] Blondel V.D., Guillaume J.-L., Lambiotte R., Lefebvre E. (2008). Fast unfolding of communities in large networks. J. Stat. Mech..

[30] Smith G.J., Walsh L., Higuchi M., Kelada S.N.P. (2019). Development of a large-scale computer-controlled ozone inhalation exposure system for rodents. Inhal. Toxicol..

[31] Patel R.K., Jain M. (2012). NGS QC toolkit: a toolkit for quality control of next generation sequencing data. PLoS One.

[32] Langmead B., Salzberg S.L. (2012). Fast gapped-read alignment with Bowtie 2. Nat. Met..

[33] Liao Y., Smyth G.K., Shi W. (2014). featureCounts: an efficient general purpose program for assigning sequence reads to genomic features. Bioinformatics.

[34] Anders S., Reyes A., Huber W. (2012). Detecting differential usage of exons from RNA-seq data. Genome Res..

[35] Huang D.W., Sherman B.T., Lempicki R.A. (2009). Systematic and integrative analysis of large gene lists using DAVID bioinformatics resources. Nat. Protoc..

[36] Huang D.W., Sherman B.T., Lempicki R.A. (2009). Bioinformatics enrichment tools: paths toward the comprehensive functional analysis of large gene lists. Nucl. Acids Res..

[37] García-Fojeda B., González-Carnicero Z., de Lorenzo A., Minutti C.M., de Tapia L., Euba B. (2019). Lung surfactant lipids provide immune protection against haemophilus influenzae respiratory infection. Front. Immunol..

[38] Dixon A.E., Que L.G. (2022). Obesity and Asthma. Semin. Respir. Crit. Care Med..

[39] Lee Y.S., Olefsky J. (2021). Chronic tissue inflammation and metabolic disease. Genes Dev..

[40] Showalter M.R., Nonnecke E.B., Linderholm A.L., Cajka T., Sa M.R., Lönnerdal B. (2018). Obesogenic diets alter metabolism in mice. PLoS One.

[41] Elliot J.G., Donovan G.M., Wang K.C.W., Green F.H.Y., James A.L., Noble P.B. (2019). Fatty airways: implications for obstructive disease. Eur. Respir. J..

[42] Mathews J.A., Krishnamoorthy N., Kasahara D.I., Cho Y., Wurmbrand A.P., Ribeiro L. (2017). IL-33 drives augmented responses to ozone in obese mice. Environ. Health Perspect..

[43] Kasahara D.I., Kim H.Y., Williams A.S., Verbout N.G., Tran J., Si H. (2012). Pulmonary inflammation induced by subacute ozone is augmented in adiponectin-deficient mice: role of IL-17A. J. Immunol..

[44] Alqahtani S., Xia L., Jannasch A., Ferreira C., Franco J., Shannahan J.H. (2021). Disruption of pulmonary resolution mediators contribute to exacerbated silver nanoparticle-induced acute inflammation in a metabolic syndrome mouse model. Toxicol. Appl. Pharmacol..

[45] Winer D.A., Winer S., Shen L., Wadia P.P., Yantha J., Paltser G. (2011). B cells promote insulin resistance through modulation of T cells and production of pathogenic IgG antibodies. Nat. Med..

[46] Ramon S., Gao F., Serhan C.N., Phipps R.P. (2012). Specialized proresolving mediators enhance human B cell differentiation to antibody-secreting cells. J. Immunol..

[47] Ramon S., Baker S.F., Sahler J.M., Kim N., Feldsott E.A., Serhan C.N. (2014). The specialized proresolving mediator 17-HDHA enhances the antibody-mediated immune response against influenza virus: a new class of adjuvant?. J. Immunol..

[48] Kato A., Hulse K.E., Tan B.K., Schleimer R.P. (2013). B lymphocyte lineage cells and the respiratory system. J. Allergy Clin. Immunol..

[49] Espéli M., Bashford-Rogers R., Sowerby J.M., Alouche N., Wong L., Denton A.E. (2019). FcγRIIb differentially regulates pre-immune and germinal center B cell tolerance in mouse and human. Nat. Commun..

[50] Hsu P.-S., Lin C.-M., Chang J.-F., Wu C.-S., Sia K.-C., Lee I.-T. (2019). Participation of NADPH oxidase-related reactive oxygen species in leptin-promoted pulmonary inflammation: regulation of cPLA2α and COX-2 expression. Int. J. Mol. Sci..

[51] Bustamante-Marin X.M., Merlino J.L., Devericks E., Carson M.S., Hursting S.D., Stewart D.A. (2021). Mechanistic targets and nutritionally relevant intervention strategies to break obesity-breast cancer links. Front. Endocrinol..

[52] Haug K., Cochrane K., Nainala V.C., Williams M., Chang J., Jayaseelan K.V. (2020). MetaboLights: a resource evolving in response to the needs of its scientific community. Nucl. Acids Res..

[53] Edgar R., Domrachev M., Lash A.E. (2002). Gene expression omnibus: NCBI gene expression and hybridization array data repository. Nucl. Acids Res..

