# Supplementary material for: Obesity reprograms the pulmonary polyunsaturated fatty acid-derived lipidome, transcriptome, and gene-oxylipin networks
Source: J Lipid Res. 2022 Aug 24;63(10):100267. doi: 10.1016/j.jlr.2022.100267 (PMC9508350; doi:10.1016/j.jlr.2022.100267)
Supplement: Supplemental Figures and Tables [file mmc1.pdf]

SUPPLEMENTAL INFORMATION:

Obesity reprograms the pulmonary polyunsaturated fatty acid-derived lipidome, transcriptome, and gene-oxylipin networks

Rafia Virk[1], Nicole Buddenbaum[1], Abrar Al-Shaer[1], Michael Armstrong[2], Jonathan Manke[2], Nichole Reisdorph[2], Selin Sergin[3], Jenifer I. Fenton[3], E. Diane Wallace[4], Brandie M. Ehrmann[4], Hannah B. Lovins[5], Kymberly M. Gowdy[5], M Ryan Smith[6,7], Gregory Smith[8,9], Samir Kelada[8,9], and Saame Raza Shaikh[1]

[1]Department of Nutrition, Gillings School of Global Public Health and School of Medicine, The University of North Carolina at Chapel Hill, Chapel Hill NC USA

[2]Department of Pharmaceutical Sciences, University of Colorado Denver Anschutz Medical Campus, Aurora CO USA

[3]Department of Food Science and Human Nutrition, College of Agriculture and Natural Resources and College of Osteopathic Medicine, Michigan State University, East Lansing Michigan USA

[4]Department of Chemistry, University of North Carolina at Chapel Hill, Chapel Hill NC USA

[5]Division of Pulmonary, Critical Care and Sleep Medicine, The Ohio State University, Columbus OH USA

[6]Division of Pulmonary, Allergy, Critical Care, and Sleep Medicine, Department of Medicine, Emory University, Atlanta GA USA

[7]Atlanta Department of Veterans Affairs Medical Center, Decatur, GA, USA

[8]Marsico Lung Institute, University of North Carolina at Chapel Hill, Chapel Hill NC USA

[9]Department of Genetics, University of North Carolina at Chapel Hill, Chapel Hill NC USA

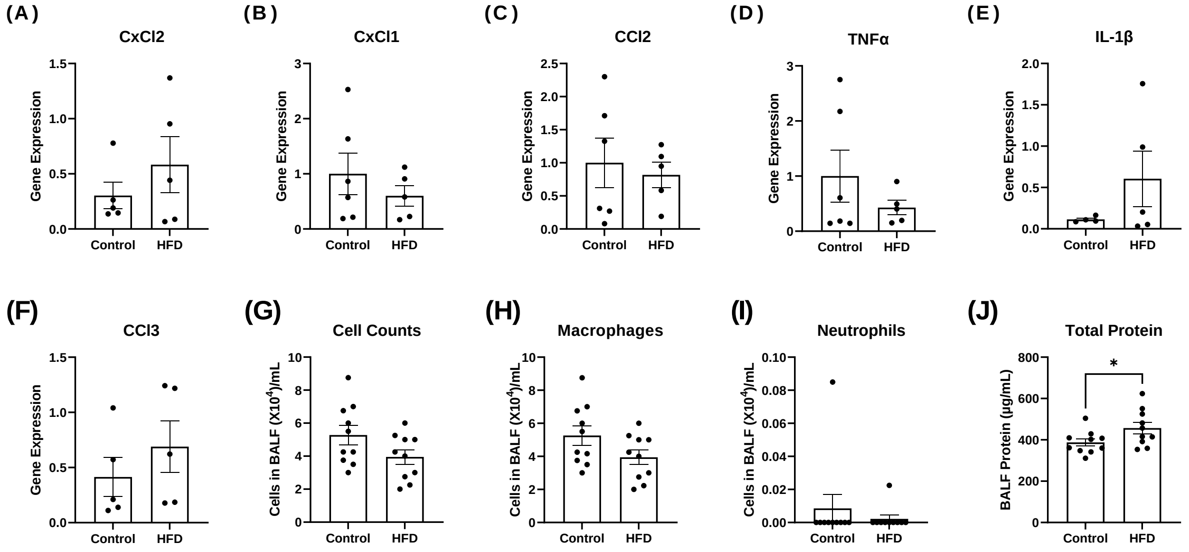

Supplemental Figure S1: Pulmonary inflammatory profile of C57BL/6 mice consuming a high fat diet. The gene expression of (A) C-X-C motif chemokine ligand 1 (CxCl1), (B) C-X-C motif chemokine ligand 2 (CxCl2), (C) CC motif chemokine ligand 2 (CCl2), (D) CC motif chemokine ligand 2 (CCl2), (E) tumor necrosis factor alpha (TNFα), and (F) interleukin 1 beta (IL-1β) was determined by real-time PCR analysis of lung tissue for C57BL/6J male mice that either consumed a lean control or a high fat diet (HFD) for 15 weeks. (G) Differential cell counts, (H) percentage of macrophages, (I) percentage of neutrophils, and (J) total protein in bronchoalveolar lavage fluid (BALF). Data are mean ± SEM from 5-6 mice per group for A-F and 10 mice per group for G-J. *$P < 0.05$ from unpaired t-test.

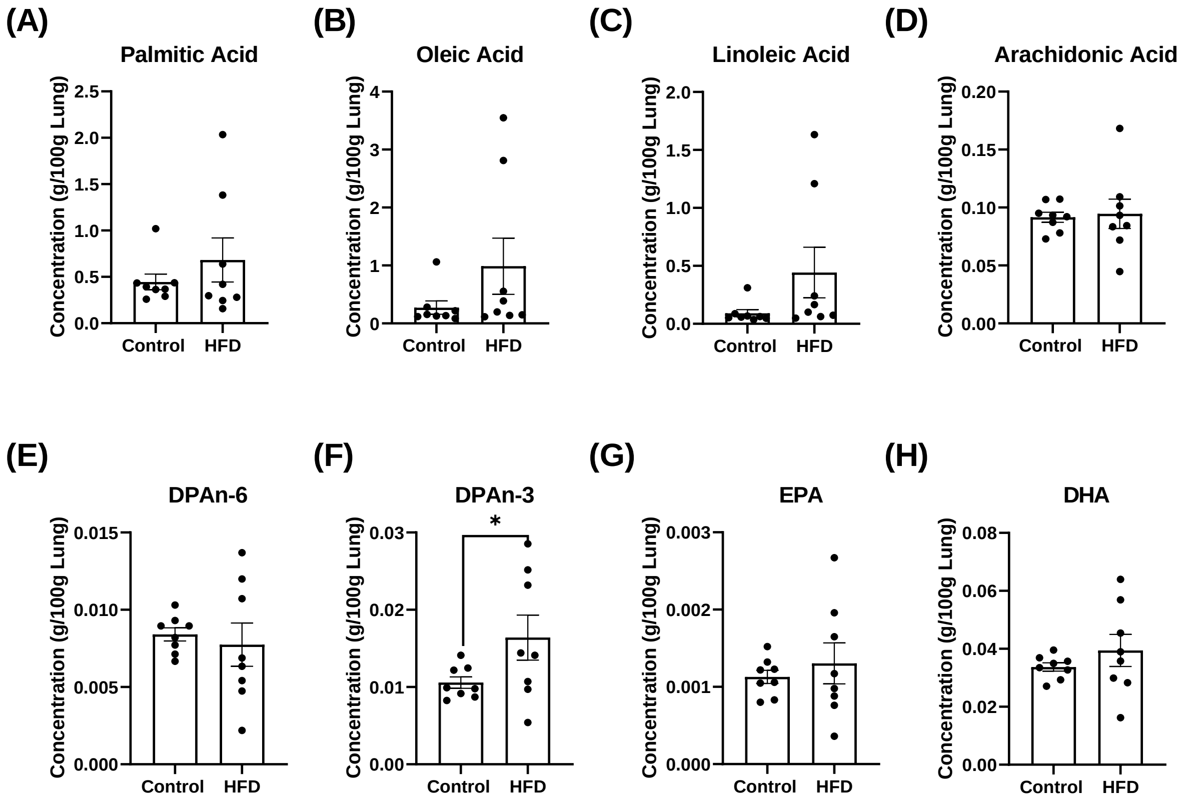

Supplemental Figure S2: The concentration of select pulmonary fatty acids is increased with a high fat diet. (A) Palmitic acid, (B) oleic acid, (C) linoleic acid, (D) arachidonic acid, (E) n-6 docosapentaenoic acid (DPAn-6), (F) n-3 docosapentaenoic acid (DPAn-3), (G) eicosapentaenoic acid (EPA), and (H) docosahexaenoic acid (DHA). C57BL/6J male mice consumed a lean control or a high fat diet (HFD) for 15 weeks. Isolated left lungs at age 21-22 weeks were used for targeted gas chromatography analysis. Data are mean ± SEM from 7-8 mice per group. *P < 0.05 from unpaired t-test.

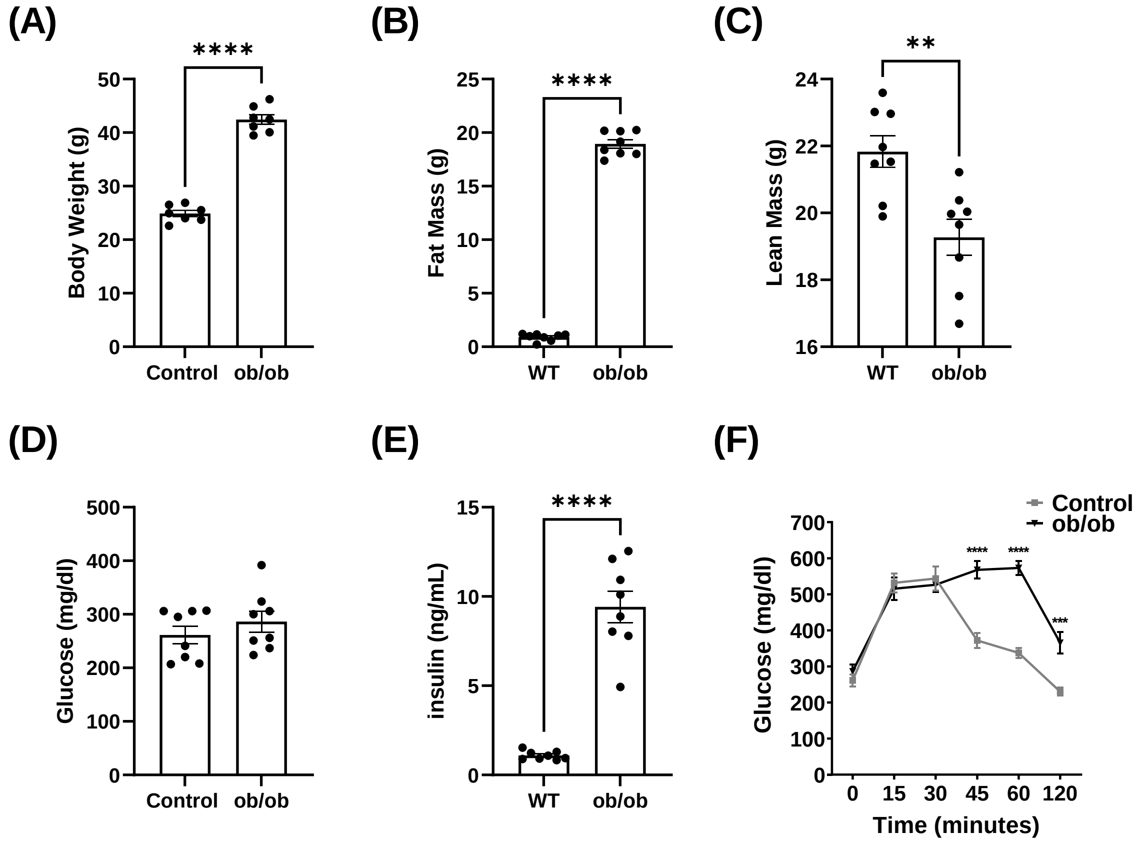

Supplemental Figure S3: Metabolic profile of a genetic model of obesity. (A) Body weights. (B) Fat mass and (C) lean mass obtained by Echo MRI. (D) Fasting glucose and (E) fasting insulin levels after a 5 h fast. (F) Glucose tolerance test completed after a 5 h fast by intraperitoneal injection of glucose. For all measurements, genetically obese (*ob/ob*) male mice and lean control mice were purchased from Jackson Laboratory at 7 weeks of age and were fed normal chow for 3 weeks. Metabolic characterization was conducted at age 10 weeks where the body weights were matched with the body weights of mice fed a high fat diet. Data are mean ± SEM from 7-8 mice per group. **P <0.01, ***P < 0.001, ****P < 0.0001 from unpaired t-test for A through E and 2-way ANOVA with Šídák's multiple comparisons test for F.

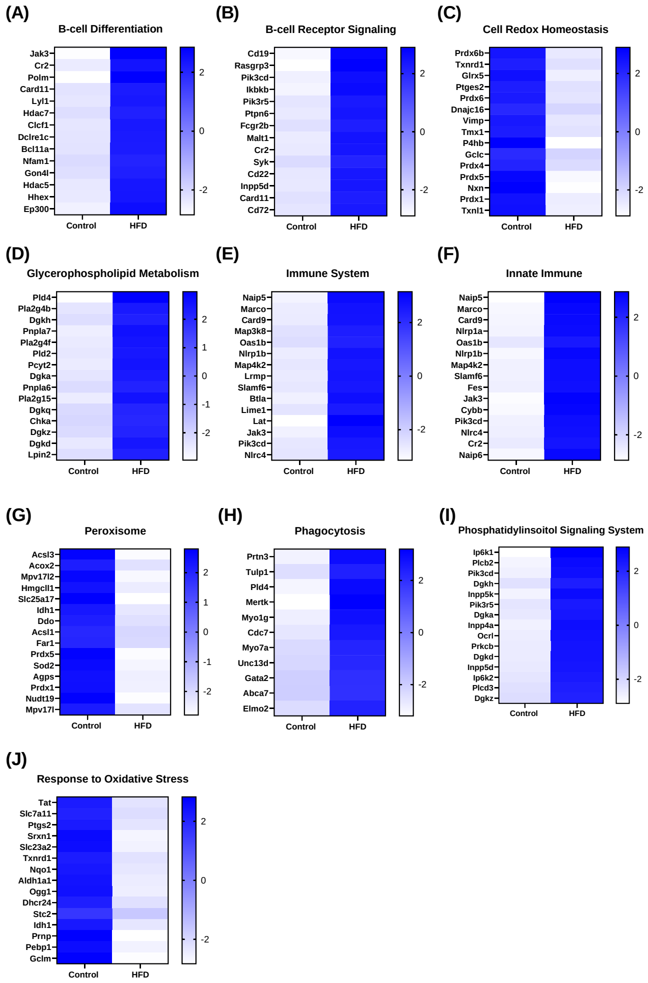

Supplemental Figure S4: Heat maps for immune-related and lipid-metabolism-related pathways and biological processes in response to high fat diet. (A) B-cell differentiation, (B) B-cell receptor signaling, (C) cell redox homeostasis, (D) glycerophospholipid metabolism, (E) immune system, (F) innate immune, (G) Peroxisome, (H) Phagocytosis, (I) phosphatidylinositol signaling system, and (J) response to oxidative stress. The heat map data are normalized to z-scores for the top 15 genes with the greatest absolute fold change and lowest adjusted p-value. C57BL/6J male mice consumed a control diet or an experimental high fat diet (HFD) for 15 weeks. N = 7-8 mice per group. All depicted pathways and biological processes are significant based on differential gene expression (DeSeq) analysis in R with Benjamani-Hochberg p-adjusted < 0.1.

Compound	Fatty acid precursor	Associated pathway	Retention time	MRM	CE	R^2	LOD (pg)	LLOQ (pg)	ULOQ (pg)	Internal Standard
Resolvin E1 (RVE1)	EPA	CYP/5-LOX	5.5	349.2>107.0	14	0.993	0.353	1.25	250	8-iso-PGF2a-d4
8-iso-15R-PGF2a	AA	ROS	7.48	353.2>193.1	16	0.991	0.34	2.5	250	8-iso-PGF2a-d4
8-iso-PGF2a	AA	ROS	7.62	353.2>193.1	16	0.995	0.717	1.25	250	8-iso-PGF2a-d4
Thromboxane B2 (TXB2)	AA	COX	7.66	369.2>168.9	18	0.993	0.103	1.25	5000	8-iso-PGF2a-d4
(11B)PGF2a	AA	COX	7.83	353.2>193.1	16	0.995	0.193	0.25	250	8-iso-PGF2a-d4
PGE2	AA	COX	8.42	351.2>315.2	8	0.998	0.126	0.25	5000	PGE2-d4
Resolvin D3 (RVD3)	DHA	15-LOX	8.48	375.2>146.9	14	0.997	0.365	0.5	250	PGE2-d4
PGF2a	AA	COX	8.51	353.2>309.2	16	0.997	0.06	0.25	5000	PGF2a-D9
Prostaglandin D_2	AA	COX	8.64	351.2>315.3	10	0.996	0.173	0.5	5000	PGF2a-D9
Resolvin D2 (RVD2)	DHA	15-LOX	8.78	375.2>141.0	13	0.995	0.793	1.25	250	Resolvin D2-d5
Lipoxin B4 (LXB4)	AA	5-LOX	8.85	351.2>220.9	14	0.995	0.614	1.25	250	Resolvin D2-d5
6α-Prostaglandin I_1	AA	COX	9.41	353.2>129.0	22	0.994	0.054	0.5	5000	8-iso-PGF2a-d4
Resolvin D1 (RVD1)	DHA	15-LOX	9.54	375.2>141.0	13	0.998	0.19	0.5	250	Resolvin D1-d5
17(R)-Resolvin D1 (17R-RVD1)	DHA	15-LOX	9.63	375.2>140.9	14	0.995	0.072	0.5	250	Resolvin D1-d5
Lipoxin A4 (LXA4)	AA	5-LOX	9.79	351.2>114.9	13	0.997	0.249	0.5	250	Resolvin D2-d5
15(R)Lipoxin A4 (15R-LXA4)	AA	5-LOX	9.81	351.2>114.9	14	0.997	0.125	1.25	250	Resolvin D2-d5
LTD4	AA	5-LOX/GST	10.47	495.3>176.9	17	0.994	0.063	1.25	250	LTD4-d5
7S Maresin R1	DHA	12-LOX	11.96	359.2>250.0	9	0.994	0.488	5	250	Resolvin D2-d5
10,17-DiHDoHE	DHA	15-LOX	12.34	359.2>153.0	13	0.994	0.245	0.25	250	LTB4-d4
LTE4	AA	5-LOX/GST	12.38	438.2>333.1	17	0.991	0.205	0.25	250	LTE4-d5
7R Maresin R1	DHA	15-LOX	12.41	359.2>250.0	17	0.997	0.908	5	250	LTB4-d4
Resolvin D5 (RVD5)	DHA	15-LOX	12.41	359.2>199.0	14	0.996	0.133	1.25	250	LTB4-d4
LTB4	AA	5-LOX	12.9	335.2>195.1	13	0.999	0.121	0.25	5000	LTB4-d4
18-HEPE	EPA	15-LOX	15.02	317.2>299.0	14	0.994	2.311	5	5000	LTB4-d4
12-HEPE	EPA	12-LOX	15.86	317.2>299.0	14	0.995	1.848	5	5000	LTB4-d4
13-HODE	LA	15-LOX	16.4	295.2>277.1	18	0.997	0.897	25	5000	9(S)-HODE-d4
13-OxoODE	LA	15-LOX	16.48	293.2>113.0	22	0.999	1.059	5	5000	LTB4-d4
9-HODE	LA	NA	16.61	295.2>277.0	18	0.998	0.934	12.5	5000	9(S)-HODE-d4
15-HETE	AA	15-LOX	16.78	319.2>219.1	9	0.997	0.545	2.5	5000	LTB4-d4
17-HDHA	DHA	15-LOX	16.82	343.2>281.2	9	0.992	3.624	5	5000	LTB4-d4

9-OxoODE	LA	NA	16.84	293.2>185.0	22	0.997*	0.163	0.25	5000	9(S)-HODE-d4
14-HDHA	DHA	12-LOX	17.12	343.2>205.0	9	0.995	0.172	1.25	5000	LTB4-d4
12-HETE	AA	12-LOX	17.4	319.2>178.7	13	0.995	0.188	1.25	5000	9(S)-HODE-d4
8-HETE	AA	ROS	17.51	319.2>154.9	13	0.997	0.605	1.25	5000	LTB4-d4
5-HETE	AA	5-LOX	18.08	319.2>115.0	9	0.998*	2.035	2.5	5000	5(S)-HETE-d8
14(15)-EET	AA	CYP	18.1	319.2>301.1	10	0.998	0.269	0.5	250	9(S)-HODE-d4
11(12)-EET	AA	CYP	18.65	319.2>178.8	10	0.995	0.089	0.25	250	9(S)-HODE-d4
Carbocyclic Thromboxane A_2	AA	COX	18.76	347.3>247.0	22	0.995	0.647	1.25	250	9(S)-HODE-d4
8(9)-EET	AA	CYP	18.86	319.2>301.1	10	0.996	0.925	5	250	9(S)-HODE-d4

Internal Standards

**Compound**	**Fatty acid precursor**	**Associated pathway**	**Retention time**	**MRM**	**CE**	**Conc. (pg/on column)**
8-iso-PGF2a-d4	AA	ROS	8.1	357.2>197.1	26	50
PGE2-d4	AA	COX	8.38	355.2>319.2	8	50
PGF2a-D9	AA	COX	8.44	362.3>318.3	21	50
Resolvin D2-d5	DHA	15-LOX	8.73	380.2>141.0	13	50
Resolvin D1-d5	DHA	15-LOX	9.48	380.2>141.0	13	50
LTD4-d5	AA	5-LOX/GST	10.42	500.3>176.8	18	50
LTE4-d5	AA	5-LOX/GST	12.34	443.3>338.2	18	50
LTB4-d4	AA	5-LOX	12.86	339.2>197.1	13	50
9(S)-HODE-d4	LA	NA	16.54	299.3>281.3	18	50
5(S)-HETE-d8	DHA	5-LOX	17.99	327.3>115.9	14	50

***These compounds use a quadratic fit; all other compounds use linear fit**

Fatty acid precursors; AA=arachidonic acid, DHA=docosahexaenoic acid, EPA=eicosapentaenoic acid, LA=linoleic acid
Associated pathway; COX=cyclooxygenase, CYP=cytochrome-p450, GST=glutathione S-transferase, 5-LOX=5-lipoxygenase,
12-LOX=12-Lipoxygenase, 15-LOX=15-lipoxygenase,
ROS=reactive oxygen species

Supplemental Table S1: MRM detection parameters and quantitation limits for all compounds, including internal standards utilizing a fixed fragmentor voltage of 380 V. All compounds demonstrated a linear calibration fit unless otherwise noted. Limit of detection (LOD), lower limit of quantitation (LLOQ) and upper limit of quantitation (ULOQ) were also evaluated.

KEGG Pathway	Count	%	PValue	Genes	List Total	Pop Hits	Pop Total	Fold Enrichment	Bonferroni	Benjamini	FDR
Protein processing in endoplasmic reticulum	42	2.281	7.53E-11	TRAM1, SAR1B, RPN1, HSPA4L, UBE2D3, UBE2D2A, HSPBP1, UBE2J1, SEC61A1, HSPH1, BAG2, LMAN2, SKP1A, UBQLN2, TXNDC5, BCAP31, XBP1, SEC13, HSPA5, AMFR, EDEM2, SSR3, DNAJB12, UBE2E2, HSPA2, RAD23B, EIF2S1, SVIP, PDIA6, CKAP4, PDIA4, DNAJC3, NPLOC4, DAD1, DNAJA2, DNAJB11, STT3A, P4HB, CRYAA, SEC24D, HSPA1B, VIMP	626	168	7691	3.071	2.09E-08	2.09E-08	2.02E-08
Metabolic pathways	160	8.691	1.73E-09	GALNT12, CHPF, UXS1, ACSM1, NAGLU, PPAT, SEPHS2, ACSL1, OXSM, ALG5, ALG2, ALG11, CMBL, MAT1A, ACSL3, PGD, PLA2G16, ALDH3A1, PGP, ACOT2, ACOT1, SUCLG2, LAP3, ALG10B, GNE, IPPK, UGT8A, NDUFB11, RPN1, ATP5J, UAP1, ADH7, AK7, AK8, LDHB, EBP, LDHA, FUT9, PPCS, ALDH3B1, RDH10, ATP5D, PGK1, ST3GAL6, HMGCLL1, MPST, GGT6, ST8SIA1, IDH1, GFPT2, GFPT1, ASNS, DHCR24, MCAT, PRDX6, PAICS, QDPR, POLA1, SQLE, GRHPR, HAL, GCLC, ADI1, CYP2S1, STT3A, ALPL, PLCH1, ALDH18A1, GALNTL6, GCLM, GALK1, GLT28D2, B4GALT1, ALAS1, PYCRL, HIBADH, TAT, PIGYL, GMPPB, SMPD3, THTPA, SPTLC1, ALDH2, SPTLC3, SMPD1, RFK, B3GALT2, AOX3, DBT, ME1, IDS, HIBCH, COX8A, CBR2, CERS3, CBR1, PCYT1B, UGT1A1, SPHK2, AKR1A1, AMPD1, PLA2G4A, NDUFC2, NME5, UGT1A6A, FH1, UGT1A6B, CYP2A5, ACOX2, TST, BDH1, DAD1, IMPAD1, ALDH1A1, ITPKA, AGPS, AGXT, TKT, FBP1, ALDH1A7, PAFAH1B2, ATP6V1B1, PDXK, OAT, PCX, NDUFB5, HDC, ODC1, HSD17B12, UQCR10, SGMS2, PTGS2, HSD17B10, PAPSS1, CKMT1, GMDS, MAT2A, HSD17B2, MGAT3, BPNT1, MGAT2, ATP6V0E2, PRPS1L3, CBR3, GALNT5, GALNT3, GCH1, TPK1, PTGES2, PTGES3, C1GALT1, MBOAT1, GCK, POLE4, CES1D, PSAT1, CES1E, MGAT4A, POLE3, UOX	626	1269	7691	1.549	4.81E-07	2.41E-07	2.32E-07
Metabolism of xenobiotics by cytochrome P450	22	1.195	1.35E-08	GSTM4, CBR2, CBR1, GSTM2, UGT1A1, GSTO1, EPHX1, GSTT3, MGST1, ADH7, UGT1A6A, UGT1A6B, ALDH3A1, AKR7A5, GSTA4, GSTA3, ALDH3B1, CYP2S1, CYP2F2, GSTM7, GSTM6, CBR3	626	64	7691	4.223	3.74E-06	1.25E-06	1.20E-06

Glutathione metabolism	18	0.978	8.61E-07	GSTM4, GSTM2, GPX2, GGT6, GSTO1, IDH1, ODC1, GSTT3, MGST1, GPX8, PGD, GCLC, GSTA4, GSTA3, LAP3, GCLM, GSTM7, GSTM6	626	55	7691	4.021	2.39E-04	5.98E-05	5.77E-05
Peroxisome	18	0.978	2.98E-04	PHYH, ACSL1, IDH1, MPV17L, MPV17L2, ACSL3, SOD2, DDO, SOD1, PRDX5, SLC25A17, ACOX2, PRDX1, AGPS, NUDT19, FAR1, AGXT, HMGCLL1	626	83	7691	2.664	7.95E-02	1.18E-02	1.14E-02
Hippo signaling pathway	26	1.412	4.49E-04	YWHAE, SERPINE1, PRKCZ, ACTG1, SOX2, PPP1CB, PPP2CA, WNT11, CCND1, YWHAQ, CDH1, YWHAH, FZD3, SMAD4, WNT7B, TRP73, FZD8, YWHAZ, PPP1CA, MOB1B, MOB1A, PPP2R2C, PPP2R2B, CTNNB1, BMPR1B, BMPR1A	626	151	7691	2.115	1.17E-01	1.56E-02	1.50E-02
Mineral absorption	11	0.598	8.51E-04	FTL1, FTH1, TRF, MT2, MT1, ATP1A1, SLC5A1, STEAP1, ATP1B1, STEAP2, SLC39A4	626	39	7691	3.465	2.11E-01	2.63E-02	2.53E-02
RNA transport	27	1.467	1.19E-03	CYFIP2, POP7, SEH1L, DDX20, RASL2-9, SUMO3, NUP62, EIF4E, RPP38, EIF2B5, RANBP2, SEC13, EIF2B2, EIF1AX, THOC3, EIF2S1, EEF1A1, EIF5, NUP50, CLNS1A, GEMIN4, NUP98, RPP25, EIF3C, EIF1A, RAN, EIF3B	626	170	7691	1.951	2.82E-01	3.01E-02	2.90E-02
N-Glycan biosynthesis	12	0.652	1.56E-03	GLT28D2, B4GALT1, DAD1, MGAT4A, ALG5, RPN1, ALG2, MGAT3, STT3A, MGAT2, ALG11, ALG10B	626	49	7691	3.009	3.53E-01	3.62E-02	3.49E-02
Sulfur metabolism	5	0.272	3.91E-03	MPST, TST, IMPAD1, BPNT1, PAPSS1	626	9	7691	6.826	6.63E-01	8.36E-02	8.06E-02

Supplemental Table S2: Downregulated Kegg Pathway enrichment results. Significantly downregulated genes based on differential gene expression (DeSeq) analysis with Benjamani-Hochberg p-adjusted < 0.1 were used for KEGG pathway generation. After KEGG pathway results were generated, only pathways with Benjamani-Hochberg p-adjusted < 0.1 are presented.

KEGG Pathway	Count	%	PValue	Genes	List Total	Pop Hits	Pop Total	Fold Enrichment	Bonferroni	Benjamini	FDR
Choline metabolism in cancer	18	1.283	1.25E-04	PDGFRA, PLA2G4F, DGKD, CHKA, SLC44A2, PRKCB, PLA2G4B, DGKA, WAS, PIK3CD, TSC1, DGKZ, PIK3R5, PLD2, MAPK10, DGKQ, RPS6KB2, DGKH	474	101	7691	2.892	3.16E-02	1.84E-02	1.72E-02
Fc gamma R-mediated phagocytosis	16	1.140	1.57E-04	SYK, MYO10, PRKCB, PRKCD, WAS, ASAP3, PIK3CD, PIK3R5, PLD2, FCGR1, INPP5D, RPS6KB2, AMPH, DOCK2, FCGR2B, LAT	474	84	7691	3.091	3.96E-02	1.84E-02	1.72E-02
Phosphatidylinositol signaling system	17	1.212	2.50E-04	DGKD, PRKCB, DGKA, PIK3CD, OCRL, DGKZ, PIK3R5, INPP4A, PIKFYVE, INPP5D, DGKQ, INPP5K, IP6K1, PLCD3, PLCB2, IP6K2, DGKH	474	97	7691	2.844	6.23E-02	1.84E-02	1.72E-02
B cell receptor signaling pathway	14	0.998	2.86E-04	CR2, CD72, SYK, PIK3CD, MALT1, RASGRP3, PIK3R5, IKBKB, INPP5D, CD19, PTPN6, FCGR2B, CD22, CARD11	474	70	7691	3.245	7.09E-02	1.84E-02	1.72E-02
Glycerophospholipid metabolism	16	1.140	5.59E-04	PLA2G4F, DGKD, PCYT2, CHKA, PNPLA7, PLA2G4B, DGKA, PLD4, TAZ, DGKZ, PLD2, PLA2G15, DGKQ, PNPLA6, LPIN2, DGKH	474	94	7691	2.762	1.34E-01	2.87E-02	2.68E-02
Transcriptional misregulation in cancer	22	1.568	1.13E-03	CSF1R, TAF15, CCNT2, CSF2, LDB1, LMO2, ASPSCR1, PAX5, TRAF1, RUNX2, ETV5, BAIAP3, FCGR1, RXRB, LYL1, HHEX, MAF, EWSR1, IL2RB, ITGB7, HIST2H3C1, GRIA3	474	165	7691	2.163	2.53E-01	4.85E-02	4.53E-02
Jak-STAT signaling pathway	20	1.426	1.36E-03	STAT5A, IL15RA, PIAS3, STAT5B, CSF2, IL15, STAT2, PIK3CD, IFNLR1, TYK2, CSF2RA, PIK3R5, LEP, IL2RB, IL3RA, IL21R, EP300, PTPN6, IL7R, JAK3	474	145	7691	2.238	2.95E-01	4.88E-02	4.56E-02
NOD-like receptor signaling pathway	11	0.784	2.22E-03	IKBKB, MAPK10, NLRP1B, NAIP5, NLRP1A, NAIP6, CARD9, NLRP3, NOD1, NLRC4, MEFV	474	57	7691	3.131	4.35E-01	6.34E-02	5.92E-02
GnRH signaling pathway	14	0.998	2.63E-03	MAP3K3, PLA2G4F, PRKCB, PLA2G4B, PRKCD, ADCY4, ADCY3, ADCY7, PLD2, MAPK10, MAPK7, GNRH1, CAMK2G, PLCB2	474	88	7691	2.581	4.92E-01	6.76E-02	6.31E-02

Supplemental Table S3: Upregulated Kegg Pathway enrichment results. Significantly upregulated genes based on differential gene expression (DeSeq) analysis with Benjamani-Hochberg p-adjusted < 0.1 were used for KEGG pathway generation. After KEGG pathway results were generated only pathways with Benjamani-Hochberg p-adjusted < 0.1 are presented.

Biological Process	Count	%	PValue	Genes	List Total	Pop Hits	Pop Total	Fold Enrichment	Bonferroni	Benjamini	FDR
cilium morphogenesis	54	2.933	1.53E-18	SPAG16, TTC26, SPEF2, TRAF3IP1, ULK4, CBY1, CDC14A, TCTN2, IQUB, TMEM107, TEKT3, NEK1, IFT57, SNAP29, DNAAF1, CCDC113, RFX3, KIF24, NME5, RPGRIP1L, WDR35, KIF27, IFT88, FAM161A, IFT81, TMEM216, WDPCP, RABL2, SSX2IP, CCNO, UNC119B, INTU, ARL6, ARL3, DYNC2LI1, IFT74, ARL13B, RAB23, TMEM67, NPHP3, TTC30A1, TTC30A2, D1ERTD622E, BBS1, IFT122, FOXJ1, PARVA, TMEM231, DNAIC2, TTC8, TMEM17, TTC30B, B9D1, ATXN10	1461	170	18082	3.931	6.18E-15	6.18E-15	6.15E-15
cilium assembly	44	2.390	1.68E-16	SPAG16, TTC26, INTU, TRAF3IP1, ARL6, CBY1, CDC14A, DYNC2LI1, IFT74, ARL13B, TMEM67, RAB23, TCTN2, TMEM107, NEK1, TTC30A1, TTC30A2, IFT57, SNAP29, BBS1, CCDC113, RFX3, IFT122, FOXJ1, KIF24, NME5, RPGRIP1L, WDR35, KIF27, TMEM231, DNAIC2, TTC8, IFT88, TMEM17, FAM161A, TTC30B, IFT81, TMEM216, B9D1, WDPCP, RABL2, ATXN10, SSX2IP, CCNO	1461	129	18082	4.221	9.00E-13	3.40E-13	3.38E-13
cell projection organization	45	2.444	2.30E-14	SPAG16, UNC119B, INTU, CCDC67, TRAF3IP1, ARL6, CCDC103, AK7, DYNC2LI1, IFT74, DCDC2A, TMEM67, TCTN2, TMEM107, IQUB, NEK1, TTC30A1, TEKT4, TTC30A2, SNAP29, D1ERTD622E, DNAAF3, CCDC113, PIFO, IFT122, FOXJ1, KIF24, PARVA, WDR35, KIF27, TMEM231, DNAIC2, TTC8, RP1, IFT88, TMEM17, FAM161A, TTC30B, IFT81, TMEM216, ARMC4, B9D1, WDPCP, SSX2IP, CCNO	1461	151	18082	3.688	9.31E-11	3.11E-11	3.09E-11
cell redox homeostasis	22	1.195	1.85E-08	TXN1, TXNDC9, PRDX6B, PTGES2, NXN, GLRX5, TXNRD1, TXNL1, DNAJC16, PDIA6, PRDX6, PDIA4, GCLC, PRDX5, PRDX4, SCO1, TMX1, PRDX1, QSOX1, P4HB, VIMP, TXNDC5	1461	65	18082	4.189	7.51E-05	1.72E-05	1.71E-05
protein folding	32	1.738	2.12E-08	TXN1, HSPA4L, TUBA1B, DNAJB6, BAG2, DNAJB4, QSOX1, CCT5, TXNDC5, CCT4, CCT3, RANBP2, RIC3, NUDC, ST13, VBP1, TXNL1, AHSA1, TBCC, PDIA6, PDIA4, CCT6A, TMX1, DNAJA4, CDC37, TCP1, DNAJA2, DNAJB11, PFDN1, P4HB, FKBP4, CRYAA	1461	128	18082	3.094	8.59E-05	1.72E-05	1.71E-05
oxidation-reduction process	93	5.052	4.44E-07	TXN1, DHRS13, PYCRL, SRXN1, HIBADH, LOXL2, CHCHD4, AKR7A5, ALDH2, IYD, FADS6, FTH1, AOX3, ME1, QSOX1, ME2, CBR2, CBR1, PHYH, GPX2, GSTO1, DIO1, AKR1A1, GPX8, NDUFC2, PGD, PTGR1, CYP39A1, ALDH3A1, POR, ACOX2, ASPH, BDH1, TMX1, ALDH1A1, AGPS, AKR1C13, BLVRB, FAR1, STEAP1, STEAP2, ALDH1A7, FTO, NXN, NDUFB11, NDUFB5, MGST1, HSD17B12,	1461	676	18082	1.703	1.80E-03	2.64E-04	2.62E-04

				UQCR10, ADH7, PTGS2, HSD17B10, DDO, LDHB, PRDX5, LDHA, PRDX4, RDH10, AGMO, PRDX1, CREG1, ALDH3B1, HSD17B2, CYP2F2, CHML, CBR3, MDH1B, CYB5B, SDR42E1, NQO1, WWOX, IDH1, TXNRD1, TXNL1, BBOX1, DHCR24, FMO3, DOHH, FAM213A, SOD2, CP, PRDX6, SOD1, HIGD2A, QDPR, SQLE, GRHPR, ADI1, CYP2S1, PIR, ALDH18A1, CYB561, UOX							
positive regulation of telomerase RNA localization to Cajal body	10	0.543	4.55E-07	CCT6A, CCT3, SHQ1, RUVBL1, TCP1, NHP2, NAF1, CCT5, NOP10, CCT4	1461	15	18082	8.251	1.84E-03	2.64E-04	2.62E-04
transport	201	10.918	2.54E-06	SLC23A2, PITPNA, AQP4, SLC4A4, ABRA, NIPAL4, CCDC91, COG8, STARD4, RBP4, RBP2, NUP98, BLZF1, SLC45A4, UNC119B, SEH1L, SLC45A3, ABCB6, SAR1B, SLC35D3, MTTP, ATP5J, SLC5A1, SLC5A3, GABARAP, SEC14L3, SCFD2, KCNMB1, TTPA, KCNMB2, ATP5D, KCNMB4, APOB, RAB11FIP4, SLC10A5, XBP1, SLC16A12, RAB39B, SLC16A11, CP, FXYD6, CLIC6, ARF4, ARF1, TRAM1, RAB7, ORM1, SLC35F3, SRPR, SLC7A11, OSBPL10, SERP1, CHMP1A, TMED2, TSPO, TMED7, SEC13, SLC2A12, SLC39A11, COMMD1, TMC5, SORCS2, VAMP8, CLDN10, RAMP3, SYPL, RASL2-9, UQCR10, SLC7A1, SLC7A2, LMAN2, ATP6V0E2, YKT6, BCAP31, RANBP2, GABRP, LRRC26, DYNLT1B, YIF1A, TMCO1, RABIF, TTC8, ESYT3, CYB561, TXN1, MTCH2, SYS1, GOLT1B, RAB3D, HDLBP, TOMM20, CHCHD4, LAPTM4B, SLC39A9, SLC16A7, TMEM38B, KPNA3, SLC39A4, KPNA1, KCNH3, SLC38A1, MMGT1, SLC30A4, THOC3, ATP1B1, STIM1, TMX1, SCNN1B, SCG5, CHMP4B, RAB38, STEAP1, STEAP2, SLC26A2, TMED10, NDUFB11, KCNE4, ARL6, ARL3, SLC1A1, SLCO1A5, SLC1A4, LIN7C, G3BP2, AP4S1, RAB6B, AP1M2, RAB4A, OSBPL3, M6PR, BSPRY, HIGD2A, 2610002M06RIK, CHMP2B, SPIRE2, IFT27, DYNLRB2, PLEKHF2, SLC48A1, OSCP1, TTC26, SFT2D3, SLC2A1, TRF, PCTP, BICD1, SEC61A1, MFSD5, NUP62, TNPO1, SNAP29, ABCC3, EIF5A, JAGN1, ABCC1, DYNLT3, NDUFC2, VPS37A, VPS37B, DDX19A, SLC25A17, CHMP3, SLC25A10, SEC22B, SLC25A5, SLC25A13, SEC22C, NDUFB5, RAB1B, GOSR2, ATP10A, SLC3A2, ATP1A1, ATP2C2, RAB25, RAB23, IGF2BP2, HNRNPA1, LRRC8E, SLC25A25,	1461	1822	18082	1.365	1.02E-02	1.29E-03	1.28E-03

				SLC25A24, SLC12A2, D1ERTD622E, TIMM8B, CYB5B, KCNJ8, TMEM30B, TXNL1, KCNIP4, LCA5, KCNJ15, GOLPH3, CLCN5, RAB15, RAB18, KCNK1, KCNK2, SLC25A33, FOLR1, RAN, SLC25A35							
ventricular system development	10	0.543	1.05E-05	BBS1, MBOAT7, ULK4, ARMC4, HYDIN, NME5, MNAT1, DPCD, AK8, KIF27	1461	20	18082	6.188	4.18E-02	4.74E-03	4.72E-03
cell-cell adhesion	34	1.847	1.96E-05	LAD1, LRRC59, TXNDC9, TES, HDLBP, TACSTD2, TWF1, SLC3A2, BZW1, FAM129B, CNN3, LDHA, BAG3, EPCAM, PRDX1, BSG, EIF4H, SFN, RPS2, LRRFIP1, EMD, YKT6, SPTBN2, NUDC, IDH1, AHSA1, PARVA, YWHAZ, PRDX6, PAICS, EIF5, VAPA, GIPC1, TAGLN2	1461	189	18082	2.226	7.65E-02	7.43E-03	7.39E-03
protein transport	78	4.237	2.02E-05	PLEKHF2, TTC26, ARF4, RAB7, ARF1, TRAM1, SYS1, SFT2D3, GOLT1B, RAB3D, TOMM20, ABRA, CHCHD4, SEC61A1, CCDC91, SERP1, NUP62, CHMP1A, TNPO1, KPNA3, SNAP29, KPNA1, EIF5A, SEC13, JAGN1, COG8, VPS37A, COMMD1, VPS37B, VAMP8, DDX19A, CHMP3, CHMP4B, RAB38, NUP98, SEC22B, BLZF1, SEC22C, UNC119B, RAMP3, SEH1L, TMED10, SAR1B, ARL6, RAB1B, ARL3, GOSR2, RASL2-9, LIN7C, GABARAP, CALCR, RAB25, SCFD2, RAB23, LMAN2, AP4S1, YKT6, RAB6B, AP1M2, D1ERTD622E, BCAP31, RANBP2, TIMM8B, RAB4A, XBP1, LCA5, RAB39B, YIF1A, RABIF, GOLPH3, TTC8, 2610002M06RIK, RAB15, CHMP2B, RAB18, SPIRE2, IFT27, RAN	1461	592	18082	1.631	7.85E-02	7.43E-03	7.39E-03
intraciliary transport	11	0.598	2.68E-05	TTC26, IFT74, TTC30B, TRAF3IP1, IFT81, ARL3, LCA5, IFT27, TTC30A1, TTC30A2, IFT57	1461	27	18082	5.042	1.03E-01	8.38E-03	8.34E-03
axoneme assembly	10	0.543	2.69E-05	SPAG16, RP1, LRGUK, RSPH1, RSPH4A, SPEF2, RSPH9, AK7, GAS8, CCDC40	1461	22	18082	5.626	1.03E-01	8.38E-03	8.34E-03
detection of calcium ion	7	0.380	4.33E-05	STIM1, KCNMB1, KCNMB2, KCNMB4, CALM3, CALM1, CALM2	1461	10	18082	8.664	1.61E-01	1.25E-02	1.25E-02
response to endoplasmic reticulum stress	18	0.978	9.35E-05	EIF2B5, XBP1, SDF2L1, HSPA5, FAM129A, THBS1, EIF2S1, PDIA6, PDIA4, UFM1, TMX1, TMEM33, STC2, UFC1, TMBIM6, P4HB, NRBF2, TXNDC5	1461	76	18082	2.931	3.15E-01	2.48E-02	2.47E-02
glutathione metabolic process	14	0.760	9.80E-05	GSTM4, GSTM2, GGT6, GSTO1, IDH1, GSTT3, MGST1, SOD2, SOD1, GCLC, GSTA4, GSTA3, GCLM, GSTM7	1461	49	18082	3.536	3.28E-01	2.48E-02	2.47E-02
epithelial cilium movement	8	0.435	1.31E-04	SPEF2, ULK4, NME5, AK7, DPCD, KIF27, GAS8, CCDC40	1461	16	18082	6.188	4.13E-01	2.98E-02	2.97E-02
response to heat	15	0.815	1.33E-04	EIF2B5, CALCA, EIF2B2, PEBP1, TACR1, HSPA2, SOD1, GCLC, CCKAR, DNAJA4, DNAJA2, CCL2, CD14, HSPA1B, LRP11	1461	57	18082	3.257	4.16E-01	2.98E-02	2.97E-02

embryonic digit morphogenesis	16	0.869	1.97E-04	SMAD4, INTU, TRAF3IP1, IFT122, GRHL2, TMEM231, LMBR1, IFT88, CHST11, RAB23, IMPAD1, TMEM107, CTNNB1, B9D1, WDPCP, BMPR1A	1461	66	18082	3.000	5.50E-01	4.20E-02	4.18E-02
epithelial cell differentiation	16	0.869	2.35E-04	TAGLN, EHF, UPK2, SIX1, DNPH1, CNN3, MUC1, UPK1A, TST, ELF3, CES1D, CASP6, TOLLIP, ANXA7, TAGLN2, CTSB	1461	67	18082	2.956	6.14E-01	4.76E-02	4.74E-02
cilium movement	11	0.598	3.21E-04	DNAIC2, RSPH4A, CCDC103, DNAAF1, ARMC4, HYDIN, LRRC6, RSPH9, DNALI1, GAS8, CCDC40	1461	35	18082	3.890	7.28E-01	5.94E-02	5.91E-02
motile cilium assembly	10	0.543	3.22E-04	DNAAF3, INTU, DNAAF1, ULK4, FOXJ1, LRRC6, RSPH9, ZMYND10, IFT57, CCDC40	1461	29	18082	4.268	7.29E-01	5.94E-02	5.91E-02
response to oxidative stress	24	1.304	3.91E-04	SLC23A2, PRNP, NQO1, ABCC1, GPX2, SRXN1, TAT, OGG1, IDH1, TXNRD1, GPX8, PEBP1, DHCR24, SLC7A11, SOD2, PTGS2, SOD1, RCAN1, GCLC, PRDX5, PRDX1, STC2, ALDH1A1, GCLM	1461	133	18082	2.233	7.95E-01	6.80E-02	6.76E-02
decidualization	9	0.489	4.03E-04	GJB2, CDH1, CTSL, STC2, BSG, LIF, PLA2G4A, PTGS2, CTSB	1461	24	18082	4.641	8.04E-01	6.80E-02	6.76E-02

Supplemental Table S4: Downregulated biological processes via gene ontology analysis. Significantly downregulated genes based on differential gene expression (DeSeq) analysis with Benjamani-Hochberg p-adjusted < 0.1 were used for gene ontology biological pathway generation. After results were generated, only biological processes with p-value < 0.001 are presented.

Biological Process	Count	%	PValue	Genes	List Total	Pop Hits	Pop Total	Fold Enrichment	Bonferroni	Benjamini	FDR
phosphorylation	79	5.631	2.16E-09	SMG1, CIITA, TRIO, MAST3, DGKA, ARAF, PIK3CD, STK19, IKBKB, STK10, FASTK, SPEG, PRKG2, MAP3K8, JAK3, MAP3K5, EPHA4, PDGFRA, MAP4K2, EPHA7, RPS6KL1, SYK, PRKCB, PRKCD, TNNI3K, TYK2, DGKZ, D8ERTD82E, FGR, DGKQ, HKDC1, ULK3, PRKD2, ALPK3, EPHA1, ALPK1, BLK, CSF1R, PFKFB4, PKN3, PAPSS2, CKMT2, DGUOK, MAPK7, MKNK1, STK38, GRK6, CSK, IP6K1, MARK3, CAMK2G, BUB1, MAP4K3, IP6K2, NTRK2, MAP3K3, HIPK4, CDK19, DMPK, CHKA, EIF2AK3, DCLK2, CDC7, EIF2AK4, MERTK, CLK4, CLK2, UCKL1, MAPK10, CLK1, CDK8, PIKFYVE, CDK5, FES, TAOK2, RPS6KB2, CDK10, PKN1, FGFR4	1144	612	18082	2.040	8.01E-06	8.01E-06	7.99E-06
intracellular signal transduction	57	4.063	1.67E-08	DGKD, MAST3, DGKA, ARAF, CBLB, NRBP2, MCF2L, RGS9, JAK3, UNC13B, MAP4K2, SYK, PRKCB, NFAM1, PRKCD, HMHA1, TYK2, GMIP, DGKZ, LAX1, RASA4, DGKQ, PRKD2, PLCB2, RAPGEF3, RAPGEF4, ARHGEF6, DGKH, RGS14, PKN3, NPR2, ADCY4, ADCY3, NOD1, RASGRP2, RASGRP1, ADCY7, MKNK1, INPP5D, STK38, DVL1, CSK, MAP4K3, SH2B1, MAP3K3, STAC3, DCLK2, MYO9B, SMAD7, PIKFYVE, NEURL2, LEP, PTPN6, PKN1, RGS11, PLCD3, LAT	1144	400	18082	2.252	6.21E-05	3.10E-05	3.09E-05
protein phosphorylation	71	5.061	9.93E-08	TRIO, MAST3, ARAF, IKBKB, STK10, NRBP2, FASTK, SPEG, PRKG2, MAP3K8, JAK3, MAP3K5, EPHA4, PDGFRA, MAP4K2, EPHA7, MORC3, RPS6KL1, SYK, PRKCB, STRADB, PRKCD, PHKA1, TNNI3K, TYK2, D8ERTD82E, FGR, ULK3, PRKD2, ALPK3, EPHA1, ALPK1, BLK, CSF1R, PKN3, NPR2, DGUOK, MAPK7, MKNK1, STK38, GRK6, CSK, MARK3, CAMK2G, BUB1, MAP4K3, NTRK2, MAP3K3, HIPK4, CDK19, DMPK, EIF2AK3, DCLK2, CDC7, GTF2H2, EIF2AK4, MERTK, CLK4, CLK2, P2RX7, MAPK10, CLK1, CDK8, PAN3, CDK5, FES, TAOK2, RPS6KB2, CDK10, PKN1, FGFR4	1144	576	18082	1.948	3.69E-04	1.23E-04	1.23E-04
immune system process	52	3.706	3.55E-07	DCLRE1C, PIK3CD, LRMP, MAP3K8, JAK3, MAP3K5, MAP4K2, CR2, SYK, DDX58, PRKCB, SP110, LAX1, LIME1, TLR1, FCGR1, FGR, NAIP5, IRF3, NAIP6, IRF1, PRKD2, TLR7, CD300C, CSF1R, NLRP1B, UNC93B1, NLRC5, AKAP8, OAS1B, NOD1, NLRC4, MEFV, SAMHD1, LY9, INPP5D, BTLA, NLRP3, CSK, SLAMF6, GSDMD, SEMA4A, CARD9, LY86, SSC5D, EIF2AK4, SERPINA3G, MARCO, TBKBP1, HC, LAT, MYO1G	1144	383	18082	2.146	1.32E-03	3.30E-04	3.29E-04
transcription, DNA-templated	162	11.547	3.48E-05	CRTC2, ZFP445, CCNK, PRDM9, ZFP444, CCNT2, HDAC10, PRDM6, UBP1, NOC2L, IKZF3, PNN, DACH1, ZFP30, SOX17, EP300, SOX8, CCNL2, ZFP579, PIAS3, TLE2, ZFP697, ZFP219, PRKCB, SP110, THOC1,	1144	1885	18082	1.358	1.21E-01	2.15E-02	2.15E-02

				HDGFRP2, RUNX2, MAF, DMTF1, EWSR1, TIMELESS, ZFP692, HOXB3, ZSCAN26, PREB, ZFP451, INO80D, NOTCH4, GATA2, HELZ2, ZFP57, ZFP182, DEAF1, ZKSCAN3, SAFB2, LPXN, NLRP3, E4F1, ZFP740, STAT5A, ZFP512, STAT5B, CBX7, ZFP511, SETDB1, BCL11A, SS18L1, PHF12, EAF2, RFXANK, POU2F2, POU6F1, SMAD7, CDK8, TFCP2, TADA2A, GON4L, SNAI2, BRWD1, RBAK, ZSCAN2, RERE, ZMYND15, MESP1, CSRNP2, RARG, CIITA, HIP1, TCF25, PHF1, AKAP8L, HIF3A, MED12, LYL1, HHEX, SCML4, SIN3B, SIX5, ZMYM5, UIMC1, HMG20A, ZFP641, CIC, ZFP280D, ZFP280C, TEAD3, BATF2, MYOCD, ZFP532, PEG3, NCOA3, DNMT3A, ZFP932, MKL1, TET1, IL16, PROX2, PAX5, SIRT7, SAFB, COMMD4, TRAF7, GCFC2, KAT2A, IRF3, TAL1, ELF4, IRF1, TFEC, MED20, RBPJL, HDAC5, SP100, SMARCD3, SFSWAP, SRRT, ZBTB48, AKAP8, CXXC1, HDAC6, HDAC7, ZFP12, RXRB, TFPT, ABLIM2, RBBP5, ATOH8, HSF4, ZFP263, PCGF1, BRD8, HES2, MLXIP, BRF1, NFYA, STAT2, GTF2H2, TBX6, ZFP354A, PER3, POLR3A, TEF, PAGR1A, TNIP2, ERCC2, PKN1, PAXBP1, TARDBP, LPIN2, ZFP276, ZFP28							
innate immune response	47	3.350	6.15E-05	BLK, CSF1R, NLRP1B, NLRP1A, UNC93B1, AKAP8, NLRC5, PIK3CD, NOD1, OAS1B, NLRC4, MEFV, SAMHD1, LY9, MALT1, NLRP3, SLAMF6, TRIM26, CSK, JAK3, MAP3K5, GSDMD, MAP4K2, CR2, SYK, SP110, DDX58, CARD9, LY86, CYBB, SSC5D, TYK2, TLR1, FGR, FCGR1, MARCO, NAIP5, POLR3A, IRF3, FES, NAIP6, ELF4, IRF1, TBKBP1, TLR7, HC, TRIM11	1144	400	18082	1.857	2.04E-01	3.26E-02	3.25E-02
regulation of Rho protein signal transduction	16	1.140	8.12E-05	ITSN2, TRIO, PLEKHG2, ARHGEF26, ARHGEF15, ARHGEF25, ARHGEF39, ARHGEF18, MYO9B, FGD6, ALS2CL, MCF2L, EPS8L1, ARHGEF1, ARHGEF40, ARHGEF6	1144	77	18082	3.284	2.60E-01	3.63E-02	3.62E-02
protein autophosphorylation	27	1.924	8.80E-05	CSF1R, SMG1, STK10, MKNK1, CSK, JAK3, CAMK2G, EPHA4, PDGFRA, NTRK2, MAP3K3, HIPK4, SYK, PRKCD, EIF2AK3, PHKA1, EIF2AK4, CLK4, CLK2, CLK1, FGR, CDK5, FES, ULK3, PRKD2, EPHA1, FGFR4	1144	183	18082	2.332	2.79E-01	3.63E-02	3.62E-02
peptidyl-tyrosine phosphorylation	14	0.998	1.81E-04	STAT5A, PDGFRA, EPHA4, CSF1R, SYK, CLK4, CLK2, CLK1, FGR, FES, PTPN6, FGFR4, EPHA1, JAK3	1144	65	18082	3.404	4.90E-01	6.73E-02	6.71E-02
peptidyl-serine phosphorylation	21	1.497	2.58E-04	HIPK4, MORC3, SMG1, SYK, DMPK, PRKCB, MAST3, PKN3, PRKCD, EIF2AK3, DCLK2, CDC7, PKD1, CLK1, MAPK7, CDK5, MKNK1, STK38, RICTOR, PRKD2, PKN1	1144	133	18082	2.496	6.17E-01	8.72E-02	8.69E-02
phagocytosis	12	0.855	3.36E-04	LEP, ELMO2, PLD4, PRTN3, CDC7, ABCA7, MYO7A, TULP1, MERTK, GATA2, UNC13D, MYO1G	1144	52	18082	3.648	7.13E-01	1.04E-01	1.04E-01

regulation of GTPase activity	14	0.998	4.50E-04	NTRK2, EPHA4, TSC1, POT1B, OCRL, RASAL3, RASGRP1, RAP1GAP, RASGRP3, RASA4, RICTOR, EPHA1, AGRN, SBF1	1144	71	18082	3.117	8.13E-01	1.29E-01	1.28E -01
B cell differentiatio n	15	1.069	5.10E-04	HDAC5, CR2, DCLRE1C, BCL11A, POLM, 5830417I10RIK, NFAM1, HDAC7, LYL1, HHEX, GON4L, CLCF1, EP300, JAK3, CARD11	1144	81	18082	2.927	8.50E-01	1.31E-01	1.31E -01
sarcomere organization	9	0.641	5.30E-04	NEURL2, ACTN2, TNNT2, TCAP, LDB3, NKX2-5, MYPN, TTN, LMOD2	1144	31	18082	4.589	8.61E-01	1.31E-01	1.31E -01
muscle contraction	11	0.784	9.88E-04	TMOD1, CHRNB1, ACTN2, TMOD4, TNNT2, SCN7A, TAZ, CLCN1, TRDN, TTN, LMOD2	1144	50	18082	3.477	9.75E-01	2.29E-01	2.29E -01

Supplemental Table S5: Upregulated biological processes via gene ontology analysis. Significantly upregulated genes based on differential gene expression (DeSeq) analysis with Benjamani-Hochberg p-adjusted < 0.1 were used for gene ontology biological pathway generation. After results were generated, only biological processes with p-value < 0.001 are presented.

Phosphoglyceride Metabolism		
**Metabolite**	**Genes (network threshold)**	
	Control	HFD
15-HEPE	No network	Was (0.74), Pik3r5 (0.87), Prkcb (0.97), Pik3cd (0.97)
19,20-DiHDPA	Chka (-0.85)	Pik3r5 (0.85), Prkcb (0.90), Pik3r5 (0.96)
11-HDHA	No network	Pik3r5 (0.87), Prkcb (0.90), Pik3cd (0.96)
14-HDHA	Prkcb (-0.76), Rps6kb2 (-0.72)	Was (0.72), Pik3r5 (0.86), Prkcb (0.90), Pik3cd (0.96)
**B-cell Receptor Signaling**		
**Metabolite**	**Genes (network threshold)**	
	Control	HFD
15-HEPE	No network	Inpp5d (0.73), Fcgr2b (0.86), Pik3r5 (0.90), Ptpn6 (0.97), Syk (1), Pik3cd (1)
19,20-DiHDPA	No network	Cd19 (-0.72), Pik3r5 (0.75)
11-HDHA	Rasgrp3 (-0.71)	Inpp5d (0.74), Pik3r5 (0.88), Fcgr2b (0.90), Syk (0.93), Pik3cd (0.96)
14-HDHA	No network	Inpp5d (0.73), Pik3r5 (0.87), Fcgr2b (0.90), Syk (0.94), Pik3cd (0.96)

Supplemental Table S6: Integration of transcriptomic and metabolomic analyses shows different network interactions for key n-3 PUFA-derived oxylipins with specific transcriptomic pathways. Differential network analysis for select n-3 PUFA derived oxylipins (15-HEPE, 19,20-DiHDPA, 11-HDHA, and 14-HDHA) was conducted for genes present in the Phosphoglyceride metabolism and B-cell receptor signaling pathways using xMWAS, a software for data integration. Here the genes are listed for each metabolite network with its network threshold value.
